# Aniline Electropolymerization on Indium–Tin Oxide Nanofilms with Different Surface Resistivity: A Comprehensive Study

**DOI:** 10.3390/nano16030165

**Published:** 2026-01-26

**Authors:** Sonia Kotowicz, Barbara Hajduk, Paweł Jarka, Agnieszka Katarzyna Pająk, Pallavi Kumari, Andreea Irina Barzic

**Affiliations:** 1 Institute of Chemistry, Faculty of Science and Technology, University of Silesia, Szkolna 9 St., 40-006 Katowice, Poland; 2 Centre of Polymer and Carbon Materials of the Polish Academy of Sciences, M. Curie-Skłodowkiej 43 St., 41-819 Zabrze, Poland; bhajduk@cmpw-pan.pl (B.H.); apajak@cmpw-pan.pl (A.K.P.); pkumari@cmpw-pan.pl (P.K.); 3 Department of Engineering Materials and Biomaterials, Faculty of Mechanical Engineering, Silesian University of Technology, Konarskiego 18A St., 44-100 Gliwice, Poland; pawel.jarka@polsl.pl; 4 “Petru Poni” Institute of Macromolecular Chemistry, Alea Grigore Ghica Vodă, No. 41A, 700487 Iaşi, Romania; cosutchi.irina@icmpp.ro

**Keywords:** polyaniline, electropolymerization, ellipsometry, ITO

## Abstract

Aniline (ANI) was electropolymerized on ITO substrates with different surface resistivities. The process was performed by cyclic voltammetry from an aqueous, homogeneous solution containing sulfuric acid and the aniline monomer using various numbers of cycles and scan rates. The resulting polymer films (PANI) were characterized by ATR-IR spectroscopy, spectroscopic ellipsometry and atomic force microscopy. The influence of ITO surface resistivity on the electropolymerization process, the quality of the obtained PANI layers, and their optical properties was evaluated. Homogeneous PANI films were produced on ITO substrates with surface resistivities of 15–25 Ω/sq, encompassing both emeraldine salt and emeraldine base forms. Although the film’s growth was rapid, it also led to adhesion issues. In contrast, for ITO substrates with surface resistivities of 70–100 Ω/sq and 80–100 Ω/sq, the resulting films showed improved adhesion but were less homogeneous. Nevertheless, the conductive emeraldine salt form of polyaniline was successfully obtained. The conductive form of polyaniline was obtained without any additional modifications to the electropolymerization procedure. Notably, the literature provides no systematic analysis of electropolymerization on ITO substrates with different surface resistivities, which opens up new research opportunities and provides a basis for the rational design and optimization of PANI-based electro-optical coatings for advanced sensing applications.

## 1. Introduction

Aniline is the simplest aromatic amine (C_6_H_5_NH_2_) in its liquid form. Freshly obtained aniline is colorless, and when exposed to air, it changes color from yellow to brown [1]. Aniline is a reactive compound that can be easily oxidized, and owing to the lone electron pair of the nitrogen atom, it has basic and nucleophilic properties [2]. The chemical polymerization of aniline was discovered as early as the 19th century. In the 20th century, the phenomenon of polymerization of this amine began to be thoroughly studied, revolutionizing the knowledge related to conducting polymers [3].

Polyaniline (PANI) is an electron conductive polymer, and the conduction of electric current occurs during its partial oxidation [4]. The electropolymerization of aniline can be carried out galvanostatically, potentiostatically and by cyclic voltammetry (with a linear cyclic change in potential in a specific potential range between the working and counter electrodes) in an acidic environment (e.g., aqueous solutions of acids), where the anodic oxidation of aniline on the electrode surface appears and subsequent chemical reactions occur [5]. The first polymerization step leads to a radical cation formation, which can be represented by four different resonance structures [5]. Next, a dimer is formed by combining two cation radicals, which is mainly p-aminodiphenylamine (PADFA), but benzidine can also be formed [6]. The next step involves the oxidation of a dimer, trimer molecules, and aniline. The resulting cation radicals can then react further to form oligomers. Moreover, through the alternating oxidation of subsequent oligomers to cation radicals and their subsequent combination into increasingly longer structures, a polymer with a linear structure, partially oxidized emeraldine is formed. The oxidation of dimers and higher oligomers occurs at lower potentials than the oxidation process of aniline [7]. Polyaniline can occur in several forms, depending on the degree of oxidation and protonation. The final products of aniline polymerization can be (i) fully reduced leuemeraldine base (LEB)—colorless, white or light yellow, non-conductive, (ii) blue semioxidized emeraldine base (EB), (iii) green conductive emeraldine salt (ES), (iv) dark blue pernigraniline salt (PAS) and (v) from dark blue to purple, non-conductive, fully oxidized pernigraniline base (PAB) [3,8,9]. It should be mentioned that the pH of the solution plays a significant role in the mechanism of aniline polymerization. The oxidation process occurs at lower potentials and slows down with decreasing concentrations of reagents for the reaction occurring in pH > 4, forming a non-conducting product [10]. In the range of pH 2.5–4, the oxidation process is very slow, the aniline monomer is protonated and the products of reaction are insoluble in aqueous media and non-conducting (oligomers with cyclic phenazine). In contrast, the monomer and imino groups in the polymer chain are protonated with the two-stage reaction at pH < 2.5, and the conducting polymer can be created [10].

The polyaniline, and other conducting polymers, have various applications, in organic electronics—OLEDs [11,12], OPVs [13,14,15,16], OFETs [17,18,19], fuel cells [20], screen printing [21], electrochromic materials [22,23] or sensors [24,25]. Polyaniline can be also used in biological applications [26], in antimicrobial therapy, drug delivery, biosensors, nerve regeneration and tissue engineering [27]. Additionally, nanocomposites containing PANI are being developed with dual functionality: conductivity and magnetism [28]. Furthermore, PANI generated heat under infrared light illumination and is employed as a component of targeted therapies—photo-theranostics [29]. In the publication [30], a series of studies related to the biocompatibility of PANI in terms of cytotoxicity and embryotoxicity were presented, showing that the greatest differences result from the chemical structure of the polymer (salt vs. base). The same research group demonstrated the behavior of embryonic stem cells on polyaniline films, demonstrating comparable cell behavior to that observed on reference tissue culture plastic [31]. The results of research on the use of polyaniline reprotonated by PAMPSA in blood-contacting devices were also presented, thanks to its conductivity, anticoagulation activity and low platelet adhesion [32]. The diverse and attractive physicochemical properties of polyaniline enable its broad application—along with its derivatives, nanocomposites, films, and blends—in the field of tissue engineering.

Although PANI electropolymerization has been widely studied, the influence of substrate properties, particularly the surface resistivity of indium–tin oxide (ITO) coatings, remains insufficiently explored. The substrate resistivity can strongly affect electropolymerization kinetics, charge-transfer processes, polymer film morphology, and the resulting optical properties. Meanwhile, spectroscopic ellipsometry has been increasingly employed to characterize the optical behavior of PANI films [33,34,35,36,37,38,39], but comprehensive studies combining electrochemical, optical, and morphological analysis on ITO substrates with different resistivities are still lacking.

In this work, we present the electropolymerization of polyaniline on glass/ITO substrates with varying surface resistivity, conducted under different numbers of cycles and scan rates, followed by optical characterization using spectroscopic ellipsometry. Additional structural and surface analyses are performed using atomic force microscopy and contact-angle measurements. A key novelty of this study is the integrated electrochemical and optical characterization of PANI layers deposited on glass/ITO with different electrical properties, providing new insight into the role of substrate resistivity in PANI film formation.

## 2. Materials and Methods

### 2.1. Materials

The aniline monomer (CAS number: 62-53-3, ≥99.5%, ρ = 1.022 g·cm^−3^) and acetone were purchased from Sigma-Aldrich, Merk, Darmstadt, Germany. Sulfuric acid (H_2_SO_4_), 95%, was purchased from POCH (Polskie Odczynniki Chemiczne S.A., Gliwice, Poland). ITO (indium tin oxide, In_2_O_3_‧(SnO_2_)_x_ with 70–100 Ω/sq (manufacturer’s data: film thickness 15–30 nm, transmittance > 87%, dimension approximately 3.7 cm × 1.2 cm × 0.11 cm), and 15–25 Ω/sq (manufacturer’s data: film thickness 60–100 nm, transmittance > 78%, dimension approximately 3.7 cm × 1.2 cm × 0.11 cm) surface resistivity on glass was purchased from Sigma-Aldrich, Merck. ITO with 80–100 Ω/sq surface resistivity on glass was purchased from Biotain Hong Kong CO (Biotain Crystal Co., Limited, Wan Chai, Hong Kong, China) (manufacturer’s data: film thickness 23 nm, transmittance ≥ 87%, dimension 5 cm × 0.9 cm × 0.11 cm). The Ag/AgCl sat. KCl reference electrode was purchased from Lambda System sp. z o.o. (Piastów, Poland). 

### 2.2. Description of the Electropolymerization Process

A transparent glass/ITO substrate before synthesis was cleaned with acetone using ultrasonic cleaner for 15 min, which was followed by water for 5 min, and after that time, it was purged with nitrogen. The electropolymerization was carried out using a three-electrode cell and cyclic voltammetry (CV) method. ITO was using as the working electrode (WE), platinum coil acted as the counter electrode (CE), and the silver wire in contact with silver chloride in a saturated KCl solution (+198 mV vs. RHE at 25 °C) acted as the reference electrode (Ag/AgCl). The aniline (liquid, light straw-colored) monomer concentration was 0.1 mol·dm^−3^. The reaction was carried out in distillated water and sulfuric acid (H_2_SO_4_) with concentration of 0.5 mol·dm^−3^. Two scan rates were used, the 50 mV·s^−1^ and 100 mV·s^−1^, and different numbers of cycles, 7 and 13 in the range of −0.3 V–1.2 V at 24 ± 1 °C. The ITO deposition active area was approximately 2 cm^2^ ± 0.09. The obtained films were dried overnight at the room temperature in a dark clean room.

### 2.3. Characterization Methods

The electropolymerization was performed using Eco Chemie AutolabPGSTAT128n potentiostat (Metrohm, Herisau, Switzerland) and a three-electrode cell. The absorption spectra of PANI on ITO 15–25 Ω/sq were registered by a HITACHI U-2900 Spectrophotometer (Minato-ku, Tokyo, Japan) and special attachment for measuring the thin layers on the glass surface at room temperature (normal incidence). The photos of the samples were taken using a Nikon objective 10X 0.21 WD = 20.3 mm (as a part of an ellipsometer equipment, SENTECH Instruments GmbH, Berlin, Germany), incident beam 1 mm. FTIR spectra were recorded on a JASCO FT/IR6700 spectrometer (JASCO Corporation, Hachioji, Tokyo, Japan) in the range of 4000–400 cm^−1^. The prepared samples were analyzed using a single-reflection diamond attenuated total reflection accessory (ATR). The water contact angle was determined using a CAM 101 optical goniometer (KSV Instruments Ltd., Monroe, CT, USA) equipped with a camera (resolution 640 × 480 pixels) at room temperature. The static sessile drop method was used to measure the water contact angle on the glass/ITO substrates. Optical characterization was performed using a Sentech SE850E ellipsometer (SENTECH Instruments GmbH, Berlin, Germany), working in the range of 240–2500 nm. The device is controlled with Spectra Ray 3 software. Here, we have used the variable-angle mode (VASE), where the measurements were taken in the range of 40–45 and 60–70 angles with the step angle of 5°. The angular range was determined experimentally due to the large degree of depolarization at angles of 50–55°; therefore, due to the same problem, the modeling range of most samples was not possible over the full wavelength range. The electrical characterization was performed using a Keithley 2635B, Signatone S-302 four-point probe at room temperature and air atmosphere. The surface morphologies of the thin films were studied by an atomic force microscope (AFM) using Park Systems XE 100 with dedicated XEI Software 5.2 Build 1 (Suwon, Republic of Korea). Data were processed and analyzed using OriginPro 2021b (OriginLab Corporation, Northampton, MA, USA).

## 3. Results and Discussion

### 3.1. Electropolymerization

The electropolymerization of polyaniline was carried out in the range of −0.3 V–1.2 V. When the process was performed at a lower potential range (for the used measuring system), the aniline oxidation signals were not recorded (no increasing current was observed in subsequent cycles). The aniline monomer oxidation (A_ox_) as an anodic signal (marked as ^a^) occurred at about E_p_^a^ = 0.8 V, and for ITO 70–100 Ω/sq, 80–100 Ω/sq and 100 mV·s^−1^, it occurred at about E_p_^a^ = 1 V (vs. Ag/AgCl) as an irreversible process. Once the cation-radical is formed, four resonance structures can exist (marked as I, II, III and IV in Figure S5) [5,40]. The process of aniline oxidation initiated PANI electropolymerization [41].

**Figure 1:**
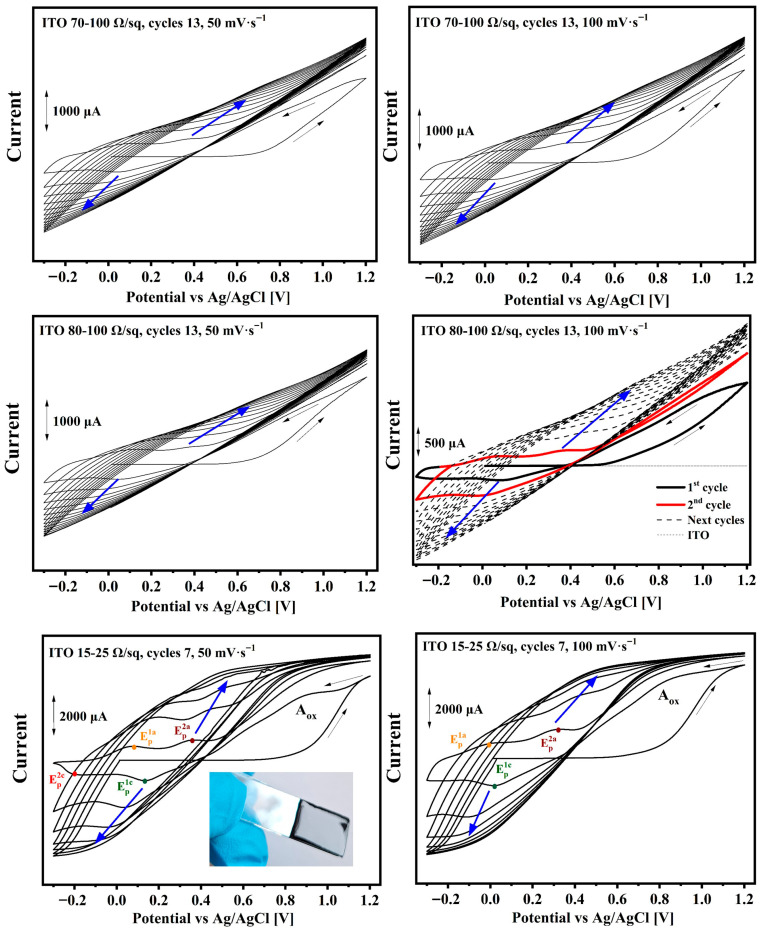
Cyclic voltammograms obtained during the electropolymerization. The process was conducted in 0.5 mol·dm^−3^ H_2_SO_4_/water solution using an ITO working electrode with different surface resistivity. The scan rates were 50 mV·s^−1^ and 100 mV·s^−1^, and 7 or 13 cycles were performed (insert: the picture of polyaniline on ITO 15–25 Ω/sq after removing from the solution).

Anodic peaks were clearly visible for ITO with 15–25 Ω/sq (Figure 1). It should be pointed out that in the first oxidation process, after switching the potential scan, the current is rising, not decreasing (Figure 1). This behavior was noticed in all presented samples. Similar behavior was also noticed by other research groups [42,43], even for polyaniline electrodeposition [44]. Furthermore, crossover of the reverse anodic scan over the cathodic indicates the nucleation phenomenon and is called a “nucleation loop” [43,44]. This process may be related to the monomer–oligomer interaction as an autocatalytic process facilitating the oxidation of the monomer [43,44]. This process is not fully understood and may have different mechanisms for different electropolymerizations.

During the reduction scan (process from 0.4 V to −0.2 V) in the first cycle, only one good-shaped cathodic peak (marked as ^c^) was seen, and the reduction of the products formed during the oxidation of aniline was noticed with variation from E_p_^1c^ = 0.01 V to E_p_^1c^ = 0.13 V. The cathodic peak shifted toward a higher potential when the scan rate was increasing (Figures S2 and S3).

The opposite behavior was observed for ITO with 15–25 Ω/sq, where for 50 mV·s^−1^, the cathodic peak was at 0.13 V, and for 100 mV·s^−1^, it was at 0.02 V. Although, in the case of ITO with 15–25 Ω/sq, for 50 mV·s^−1^, a second peak was detected at approximately E_p_^2c^ = −0.2 V. After aniline oxidation (A_ox_), in the next step, the reaction between radical cations I and III (presented in Figure S4) leads to the formation of the dimer PADPA—*p*-aminodiphenylamine, and this reaction occurs at potentials below the oxidation potential of aniline. In subsequent steps, through oxidation of the dimer and oxidation with another aniline radical cation, the polymer chain can grow in a linear form. However, when the reaction is carried out at higher potentials, a larger amount of cation radicals can be generated, and reactions between them become more likely, which may lead to the formation of phenazine units as well as cross-linked structures [5].

Such behavior was observed at potentials above 0.8 V by Professor Łapkowski’s group, where two middle peaks were detected in the subsequent anodic process [45]. In contrast, during the polymerization process described in this paper, no such peaks are observed.

According to the literature, the aniline oxidation process occurs at a higher potential than for other polyaniline structures [40]. This was also confirmed in these investigations, because two anodic peaks in the second cycle were recorded at a lower potential (Figure 1 and Figure S1). In many works, two reduction and two oxidation peaks were revealed [46,47,48,49,50,51]. These anodic signals can be assigned to the oxidation of leucomeraldine (non-conducting PANI form, LEB) (from E_p_^1a^ = 0.005 V to E_p_^1a^ = 0.15 V) converted into emeraldine (EB) form [40,49]. The subsequent peak from E_p_^2a^ = 0.31 V to E_p_^2a^ = 0.38 V can be attributed to the oxidation of emeraldine to pernigraniline (PAB), which is the fully oxidized and non-conducting form of polyaniline (PANI) (Figure S5) [52,53]. During the polymerization process, the change in color was noticed as the potential increased as follows: light yellow → green → dark green (a little bit of blue) (Table S1). Korent’s group observed a splitting of the first cathodic peak, indicating the formation of *p*-benzoquinone as a result of hydrolysis of the imine bond to quinoid rings (with Au as the working electrode and Ag as the QRE) [49]. In our work, no splitting of the cathodic peaks was observed; instead, one or two cathodic peaks were detected, corresponding to the reduction of pernigraniline to emeraldine and subsequently emeraldine to leucoemeraldine [44]. Okamoto et al. demonstrated that the formation of emeraldine salt (the conductive, green form of PANI) occurs at pH values below 1.7, whereas an increase in pH also promotes the formation of oligomers [54]. In the work of Sapurina’s group, it was reported that for PANI polymerization at pH values below 2.5, the initial stage of polymer growth occurs at low potentials, and during this stage, the intermediate product of aniline polymerization, phenazine, can be formed via electrophilic substitution [10]. It was also mentioned that the chain growth of PANI, due to a high energetic barrier, may occur at higher potentials, around 1.05 V, which is also consistent with phenazine initiation [10]. It was also shown in another work that the decrease in pH during the polymerization reaction favors the formation of a tail-to-tail aniline dimer—benzidine [55]. However, as already mentioned, no additional peaks were recorded during oxidation and reduction in the studies presented in this work. In subsequent cycles, a shift and increase in current were observed for the cathodic and anodic peaks (Figure S4). The correlation coefficient has mostly better current fitting for cathodic peaks than for anodic ones, and the same relationship was observed for E_p_ (Figures S2–S4). Beyond the fourth cycle, the first anodic peak was not seen, and the shape of voltammograms was far from rectangular (Figure 1 and Figure S1). The absence of a well-defined first anodic peak and second cathodic peak may be attributed to the limitations in charge transport within the growing polymer layer, which could hinder signal detection within the electrochemical potential window employed in this study. The distinct formation of anodic and cathodic peaks may also be influenced by the active surface area of the electrode (i.e., the surface on which the electropolymerization process takes place) (cf. discussion below). The voltammograms with a similar shape have been registered by Lyu Hailong, but the paper did not provide information about ITO surface resistivity [41]. A different structure of the voltammograms was recorded for ITO with 15–25 Ω/sq. At approximately 0.7 V, no current increase was observed, but a smoothing of the curve was, and above the 6th cycle, no changes were seen in the anodic and cathodic signals, and the shape of the voltammogram did not change. Furthermore, no changes in shape or peak shifts were observed for PANI deposition even for 13 cycles; only a dark green product (with even blue) was visible (Figure 1). Taking into account the ATR-IR analysis (cf. Section 3.2), the color of the obtained PANI (Figure S1), the shape of the voltammograms (Figure 1), and literature data, it can be concluded that oxidation of the leucoemeraldine base initially leads to the formation of the emeraldine base, which contains both benzenoid (B) and quinoid (Q) rings within the polymer chain (Figure S5). Upon protonation in the presence of sulfuric acid (VI), the emeraldine base is converted into the emeraldine salt (green form). This process occurs sufficiently rapidly so that the characteristic blue color of the emeraldine base was not observed during polymerization; instead, the green color typical of the emeraldine salt was detected directly. During the reduction process, both the emeraldine base and emeraldine salt may be reduced to their corresponding leucoemeraldine forms (LEB and LES). The coexistence of all four PANI forms cannot be excluded. However, the emeraldine salt is the predominant form of the obtained polymer (cf. Section 3.3 and Section 3.5). Therefore, it can be concluded that the deposition of PANI on ITO with 15–25 Ω/sq occurs very quickly, with well-developed anodic and cathodic peaks, and after seven cycles, a visually uniform thick layer of polyaniline was obtained. However, the obtained layers turned out to be not resistant to mechanical damage and could be easily removed from the ITO surface; overnight drying resulted in enhanced mechanical durability of the layers in the case of 7 cycles. Nevertheless, after 13 cycles, a problem with the adhesion to the substrate (between PANI and ITO) was clearly visible, and later, after overnight drying, the obtained PANI separated very easily from the ITO, which prevented further testing and analysis. The mass and thickness of the obtained polymers for ITO with 15–25 Ω/sq were estimated for the last voltammetric scan, and equations based on [41,56,57] are presented in the Supplementary Materials. The calculations were performed assuming continuity and uniformity of the obtained layers. Although, it should be kept in mind that an accurate determination of the polymer mass deposit by cyclic voltammetry might be difficult [58], and the calculations assumed 2.5 electrons per monomer [59,60]. PANI mass was calculated for the scan rate of 50 mV·s^−1^ at about 27 µg and about 19 µg for a scan rate of 100 mV·s^−1^. An increase in the electropolymerization scan rate to higher values resulted in a decrease in the thickness of the polyaniline layer from approximately 12 µm for 50 mV·s^−1^ to about 10 µm for 100 mV·s^−1^. Specific and areal capacitances were also calculated, which were dependent on the previous mass calculations ([58,61], Supplementary Materials). Polyaniline has found broad application in energy storage and as an electroactive material, owing to the pseudocapacitive behavior arising from multiple oxidation states [62]. The areal capacitance (C_ar_) at about 33 mF·cm^−2^ was calculated for a lower scan rate, and that at 28 mF·cm^−2^ was calculated for 100 mV·s^−1^. This same tendency was noticed for specific capacitance (C_sp_), which decreased as the scan rate increased (from 997 F·g^−1^ to 1000 F·g^−1^). The areal capacitance was lower for 100 mV·s^−1^ for all of the investigated ITO; however, the differences were minor (from 11 mF·cm^−2^ to 6 mF·cm^−2^, Table S2). In the case of the specific capacitance, the tendency was opposite; moreover, the C_sp_ was similar for ITO 70–100 Ω/sq and 80–100 Ω/sq. The higher specific capacitance (~1000 F·g^−1^) observed for PANI deposited on ITO with a lower sheet resistance (15–25 Ω/sq) can be attributed primarily to the higher mass loading of polyaniline formed under these conditions. As determined experimentally, the mass of PANI deposited on ITO 15–25 Ω/sq was significantly higher than that obtained for ITO substrates with sheet resistances of 70–100 Ω/sq and 80–100 Ω/sq. The lower sheet resistance of the substrate facilitates more efficient charge transport during electropolymerization, promoting thicker and more electrochemically active PANI films. Consequently, the increased availability of redox-active sites contributes to the higher specific capacitance values. In contrast, thinner PANI layers deposited on higher-resistance ITO substrates exhibit lower mass loading and reduced pseudocapacitive contribution, resulting in specific capacitance values in the range of 515–605 F·g^−1^. Moreover, an increase in the scan rate resulted in a decrease in the specific capacitance, which can be attributed to the limited time available for ion diffusion into the polyaniline chains, thereby restricting the interfacial charge storage [63]. The reported specific capacitance values for PANI and PANI composites in the literature vary widely, ranging from 70 F·g^−1^ to 2136 F·g^−1^, depending on the electrode architecture and electrolyte composition [62]. The highest specific capacitance was reported for PANI/graphene in 6 M H_2_SO_4_ [64]. The values obtained in this work are comparable to those reported for PANI deposited on Ni foam with an active area of 1 cm^2^, where a specific capacitance of 551 F·g^−1^ was achieved [63]. Higher specific capacitance values (up to 757 F·g^−1^) were reported by Shaikh et al. for PANI deposited on stainless steel substrates [65]. Overall, the electrochemical performance of polyaniline is governed by multiple factors, including the degree of doping, effective electrode surface area, film morphology, ion diffusion kinetics, and the electrode–electrolyte interface [62].

The non-uniform layers were obtained for the remaining ITO substrates (Table 1 and Tables S1). At the interface (solution–air), a dark green product was visible, followed by green conductive emeraldine in the middle, and for ITO with 80–100 Ω/sq, at the end, a dark green layer and even blue was also seen (Table S1).

Many scientists have obtained uniform layers for smaller deposition areas (≤1 cm^2^) than for the surface presented in this paper (≈2 cm^2^) [49,66]. The lack of uniformity of the layer may result from disturbances in the current flow between the point of contact of the ITO electrode with the wire (connection point) and the most immersive part of ITO (as already mentioned). At the solution–air interface, the current reaches quickly, and therefore, the aniline oxidation process occurs easily, and the products already formed on the ITO surface hinder the flow of current into the immersed substrate. Therefore, green emeraldine can be observed in the central part, as was mentioned above.

### 3.2. ATR IR Analysis

ATR IR spectra were recorded for the central, green part of the glass/ITO/PANI, and the selected spectra are shown in Figure S6. For PANI obtained on ITO 80–100 Ω/sq and 70–100 Ω/sq, distinct broad signals were observed at approximately 3360 cm^−1^ and in the range of 3299–3216 cm^−1^ corresponding to the –NH–stretching vibrations and –NH= signals of emeraldine, respectively [67]. In the case of PANI on ITO, 15–25 Ω/sq absorption peaks in this range are poorly developed. The –C–H stretching vibrations were noticed at around 2915 cm^−1^ and 2840 cm^−1^ of asymmetric and symmetric signals from polymerized benzenoid (B) [67,68]. The absorption bands at 1586–1541 cm^−1^ belong to the –C=C– stretching signals of the quinoid (Q) ring and those at 1503–1484 cm^−1^ belong to the –C=C– stretching signals of the benzenoid (B) ring [69]. A small but distinct signal was also visible approximately at 1288 cm^−1^, which can be attributed to the C–H bond signals in B and asymmetric signals of C–H in Q [68]. A wide, clear, well-developed absorption band was observed in the range of 1147–1134 cm^−1^ of the vibrations in the –NH= structure (from the protonation of nitrogen atoms) or the polaron lattice in B–N^+‧^H–B [70,71]. Splitting this signal into two, weak peaks of PANI on ITO 15–25 Ω/sq can suggest the existence of the –N^+^H= structure from the PANI base [67]. The second, well-developed signal is the absorption signal at about 1023 cm^−1^, which can be assigned to the S=O signals, and the signal at 690 cm^−1^ can be assigned to the S–O vibrations [67,72]. A band at around 860 cm^−1^ is also observed and may be allocated to the out-of-plane deformations of C–H in 1,4-subtituted rings [70].

### 3.3. Absorption Study

The absorbance spectra were obtained from optical transmittance measurements performed with the ellipsometric transmission mode. The polyaniline layers deposited onto ITO substrates were not uniform; due to this fact, the partially transparent place of the layers (inside the green part) were chosen for the measurement. The obtained curves are presented in Figure S7a. Two wide distinct absorption bands can be observed in the range of 300–600 nm (λ_abs_ in the range 352–430 nm, 3.52–2.88 eV) and 660–1200 nm (around 1.51–1.56 eV) (Table S3). In publications, it can be found that π–π* transitions are assigned in the range of 315–330 nm [49,73,74]. As the measurements were conducted in air, the partial oxidation of polyaniline cannot be excluded, which may result in the presence of the emeraldine salt form [47,49].

**Figure 2:**
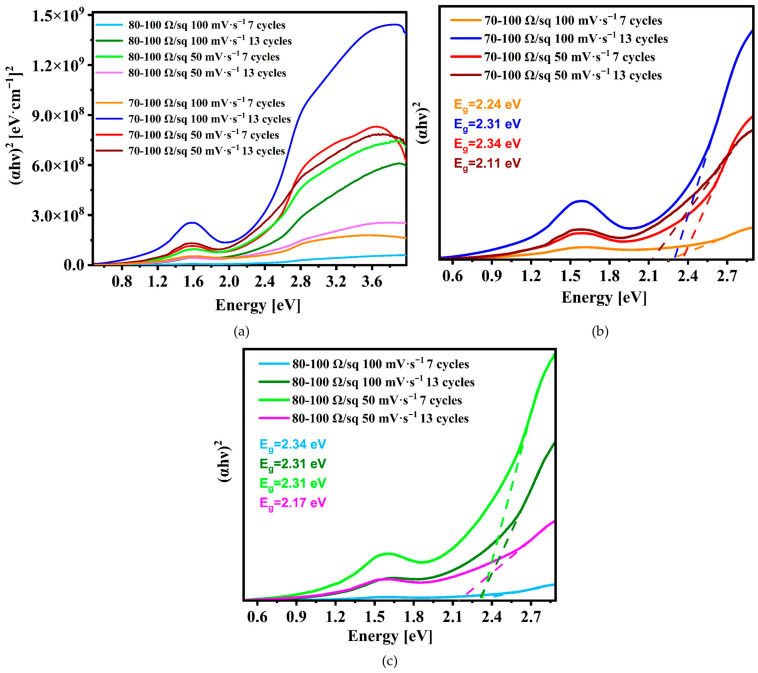
(**a**) The Tauc plot of PANI with energy band gaps of PANI deposited on (**b**) ITO 70–100 Ω/sq and (**c**) ITO 80–100 Ω/sq.

For PANI deposited on ITO 70–100 Ω/sq, an absorption band at approximately 420 nm was observed (also for ITO 80–100 Ω/sq, 7 cycles and 50 mV·s^−1^) (Figure 2a and Figure S7a). This maximum can be attributed to the diphenyl-*p*-phenylenediimine or the polaron-π* transition [49]. At approximately 600 nm, an increase in absorption was observed with the maximum recorded in the range of 794–821 nm. The λ_abs_ in this range indicates that the conducting form of emeraldine salt (ES) has been obtained, and the given maximum can be attributed to the π–polaron transition [49,70]. The position of this band is dependent on the degree of doping of polyaniline: the higher the doping, the more the band is shifted toward lower energies [49]. In the case of PANI deposited on ITO with a sheet resistance of 15–25 Ω/sq, a reliable transmission spectrum could not be obtained due to the excessive thickness of the layer. However, a UV–Vis spectrum was successfully recorded (Figure S7b), as the measurement was performed with the incident beam oriented normal to the sample surface. An additional absorption maximum at 674 nm (1.84 eV) was observed in the spectrum. This maximum is probably due to the partial deprotonation of the emeraldine salt and the partial presence of the emeraldine base (cf. Section 3.2) [73,75]. The emeraldine base absorbed at approximately 600 nm due to the intramolecular charge transfer exciton [76]. The energy gaps (E_g_) were obtained using the graphical Tauc method. Because in the case of PANI, the most dominant are local and strongly allowed transitions (π–π* and polaron–π *), which is physically most similar to the assumptions of direct transitions described by the Tauc method [77,78,79,80]. The relation (αhν)^2^ vs. (hν) was used. All the obtained curves are presented in Figure 2b,c. For the absorption calculations, we have used the thickness values obtained using the ellipsometry (presented in Table 3 in a later section of this paper). Due to the different intensities of the curves, originating from variations in sample thickness, the energy gaps were determined by graphical extrapolation, as shown in Figure 2b,c. Based on the obtained results, it can be concluded that the energy gap values determined for PANI are in the range of approximately 2.11–2.34 eV. The observed variations are relatively small, indicating that modifications of the electrodeposition parameters do not alter the absorption mechanism itself but rather influence the degree of ordering and doping of PANI. It is evident that an increased number of deposition cycles leads to a reduction in the energy gap. This effect can be attributed to the improved delocalization of electronic states and a higher contribution of polaronic states in thicker layers. Moreover, higher energy gap values observed for a smaller number of cycles and higher scan rates may indicate the presence of shorter, less conjugated chain segments and a lower degree of polyaniline doping.

### 3.4. Spectroscopic Ellipsometry

#### 3.4.1. Applied Ellipsometric Models

First, clean ITO substrates were measured and divided into three types: Type 1—ITO 70–100 Ω/sq, Type 2—80–100 Ω/sq and Type 3—15–25 Ω/sq. The model applied for ITO types 1–3 consisted of the three layers (microscopic glass/ITO/air) presented in Figure S8.

**Figure 3:**
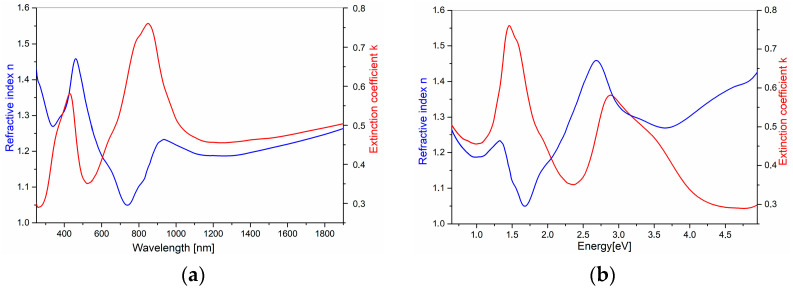
Refractive index and extinction coefficient in (**a**) wavelength scale and in (**b**) energy scale of PANI on 80–100 Ω/sq, 13 cycles and 50 mV·s^−1^.

The microscopic glass was fitted using the Cauchy model [81,82], the ITO layer with three Drude–Lorentz oscillators [83,84] and the air with the file layer. In the next step, the polyaniline films were measured. The model used for these samples consisted of four layers, where the polyaniline films were fitted using the file layer (point by point fitting), as presented in Figure 3b. The optical and dielectric coefficients were obtained for ITO types 1–3. The refractive indices and extinction coefficients are shown in Figure S9a,b, while the real and imaginary parts of the dielectric function are presented in Figure S9c,d. The values of these coefficients taken at the wavelength λ = 1900 nm are listed in Table S4. While the extinction coefficient (Figure S9a) remains low in the visible range, the refractive index (Figure S9b) gradually decreases as the wavelength increases. This property is characteristic of thin ITO films, which exhibit high transparency due to electromagnetic radiation. Around the wavelength λ = 1200 nm, the extinction coefficients begin to increase, which is associated with enhanced absorption by free carriers. At the same time, for the determined dielectric functions, it can be observed that the real part—ε_1_ (Figure S9c) shows a steady decrease, which is a typical effect when this function is modeled using the Drude model and results from the strong contribution of conduction electrons to the optical response. The imaginary part of the dielectric function (Figure S9d) displays a pronounced peak in the shorter wavelength range. Since the imaginary part ε_2_ corresponds to light absorption in the material, this indicates an enhanced absorption of the light beam in this region, accompanied by a simultaneous loss of energy, which is transformed into the material’s internal, and consequently thermal, energy. The values of the optical coefficients taken at λ = 1900 nm, along with the determined thickness values, are listed in Table S5. As it was shown in the experimental section, the ITO films were fit with the Drude–Lorentz model. The parameters—Drude plasma frequency, Ω_p_, and Drude attenuation coefficient/electron scattering frequency, Ω_τ_—and their thickness, obtained experimentally in this way are shown in Table S5. Using the Drude parameters included in Table S5, the so-called plasmon frequency can be calculated. This is the material’s free charge carriers’ oscillation frequency, which shows how strongly the electron gas reacts to an electromagnetic field. The following formula was used for the calculation of plasma frequency:

$$\omega_{p}=2\pi c\cdot \Omega_{p}$$

where *c*—is the speed of light (*c* = 3·10^8^ m/s) and *Ω_p_* [1/cm] is the Drude plasma frequency, which was determined ellipsometrically. Having the plasma frequency, we can determine the carrier concentration *N*, using the know formula below:

$$\omega_{p}=\sqrt{\frac{Ne^{2}}{\varepsilon_{0}{m_{e}}^{*}}}\to N=\frac{\omega_{p}^{2}\varepsilon_{0}{m_{e}}^{*}}{e^{2}}$$

where *N* is the carrier concentration, *ε*_0_ is the dielectric permittivity, which is equal to 8.85·10^−12^ F/m, e is the electric charge equal to 1.602·10^−19^ C and $m_{e}^{*}$ is the effective mass of free electron in ITO; in this case, we take the assumption $m_{e}^{*}\approx m_{0e}$ [85], where $m_{0e}$ = 9.11·10^−31^ kg.

The lifetime of electrons *τ* can be obtained from a simple equation:

$$\tau =\frac{1}{\omega_{\tau }}=\frac{1}{\Omega_{\tau }\cdot c}$$

The carrier mobility *μ* can be determined from the following equation:

$$\mu =\frac{e\cdot \tau }{m_{e}^{*}}$$

The conductivity *σ* is calculated from Drude parameters and the surface resistance *R*:

$$\sigma =\frac{\varepsilon_{0\cdot }\omega_{p}^{2}}{\omega_{\tau }}\to \;R=\frac{1}{\sigma \cdot d}$$

where *d* is the ITO film thickness.

The results obtained using the Drude model, presented in Table 2, are in good coincidence with the parameters declared by the ITO supplier, which only in one case (ITO type 2) slightly differ from the declared values.

#### 3.4.2. Analysis of Results Obtained for the Polyaniline Films

The films of polyaniline doped with H_2_SO_4_ acid were obtained electrochemically on the ITO substrates, whose detailed analysis is discussed above. For obvious reasons, this technique is effective, but it does not allow the formation of perfectly smooth and fully uniform layers. Additionally, due to the high light absorption of the resulting films, some of them could not be subjected to the ellipsometric measurements (PANI on 15–25 Ω/sq). For this reason, the measurement spot for each sample was selected very precisely, i.e., in a region where the layer was relatively uniform and the light absorption was not excessively strong. The obtained results showed a high degree of depolarization of the samples in the range from 790 to 2500 nm as well as pronounced oscillations visible above this range. Even though some of the spectra were smoothed, fitting the theoretical curve did not yield satisfactory agreement across the entire wavelength range. Therefore, in order to present the dispersion curves of the optical coefficients (k, n) and dielectric coefficients (ε_1_, ε_2_), one representative sample (PANI on 80–100 Ω/sq, 13 cycles and 50 mV·s^−1^) was selected, for which the spectral shape was similar to the literature data [86], and the smallest mean square error (MSE) was obtained. These results are shown in Figure 3a,b.

As it is visibly shown, the PANI polymer exhibits several distinct maxima in both optical coefficients—n and k, which correspond with characteristic electronic transitions within the polymer structure. A strong maximum in n and k appears in the range of approximately 300–500 nm—this is typical of the π–π* transition in the benzenoid form of polyaniline [49]. A second, broad maximum (around 700–900 nm) typically indicates polaronic and bipolaronic transitions, which are characteristic of the doped (protonated) form, i.e., the conducting polyaniline (emeraldine salt) [49,70]. Both coefficients with increasing wavelength (above ~1200 nm) become more stable, suggesting limited absorption in the near-infrared region.

**Figure 4:**
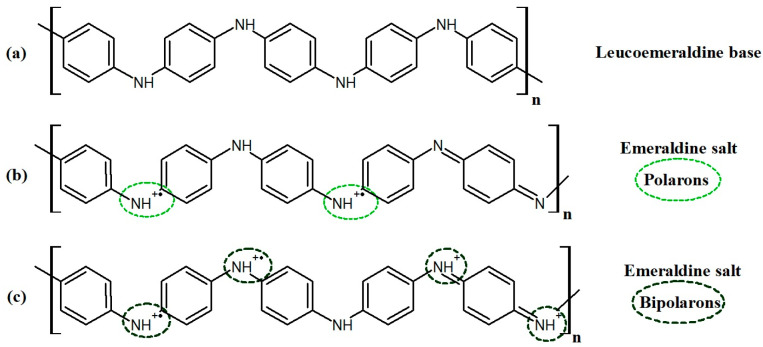
(**a**) PANI chemical structure and (**b**) polaronic and (**c**) bipolaronic states in emeraldine.

**Figure 5:**
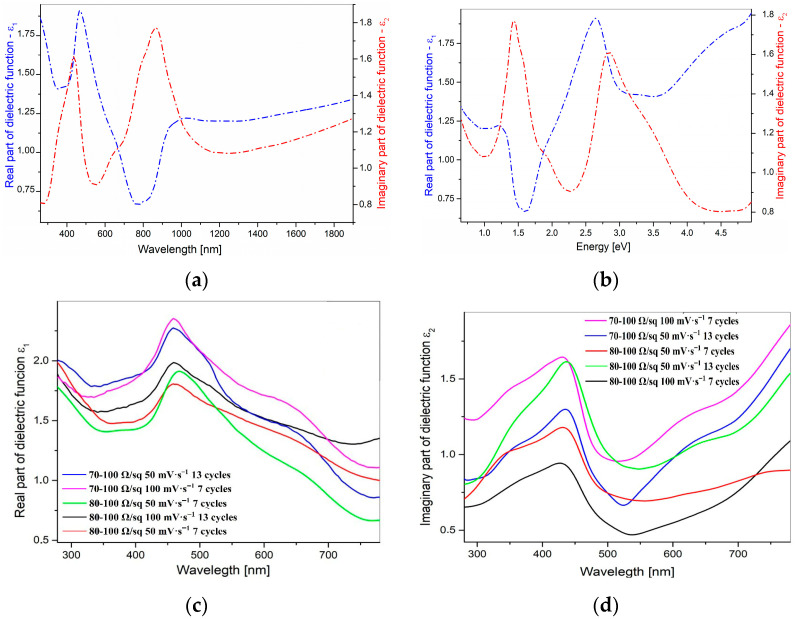
(**a**) Real and (**b**) imaginary part of dielectric function of PANI on 80–100 Ω/sq, 13 cycles and 50 mV·s^−1^ in (**a**) the wavelength scale and in (**b**) the energy scale and (**c**) real and (**d**) imaginary parts of the dielectric function in the wavelength scale for the rest of the samples.

The material absorbs strongly in the UV–Vis spectrum, which is typical for the conducting polymers with a delocalized π system. The presence of visible absorption peaks confirms the formation of polaron/bipolaron states—crucial for PANI conductivity [86,87]. Both types of these states are presented in Figure 4. The real and imaginary part of dielectric function is presented in Figure 5. The real part (ε_1_) reaches its highest values in the spectral range associated with intense electronic transitions, which indicates the strong polarizability of the material. The imaginary part (ε_2_) is pronounced with two electronic bands, which is similar to extinction coefficient k. The high values of ε_1_ and ε_2_ in the polaron transition regions reflect the strong interaction of the polymer structure with electromagnetic radiation and confirm the conductive nature of PANI. The remaining results present the optical coefficients, n and k, in Figure S10.

The PANI films deposited on ITO 3 type showed significant surface roughness (which was not visible to the naked eye, even after drying) and extremely strong optical absorption as well as a thickness beyond the ellipsometer’s measurement range.

In all the cases, we can observe the features characteristic for the doped PANI polymer. The broad absorption band in the range of 280–500 nm originated from the π–π* transitions and the polaronic transition with a band maximum at around 410–438 nm, as was mentioned earlier (cf. Section 3.3). The different intensities of these bands are coming from the different optical densities and different thicknesses of the films (which are presented in Table 3).

**Table 3:** Thickness values of PANI determined ellipsometrically.

Number of Cycles/Scan Rate	Polyaniline Thickness d [nm] on ITO with:		
	70–100 Ω/sq	80–100 Ω/sq	15–25 Ω/sq
7/50 mV s^−1^	360	488	-
13/50 mV s^−1^	745	685	-
7/100 mV s^−1^	244	340	-
13/100 mV s^−1^	389	615	-

The ITO differs in terms of carrier mobility and electron mobility. Their conductivity values are well correlated with the carrier mobility and plasma frequency. It was expected that the physical parameters obtained for ITO substrates will be significantly correlated to the electropolymerization rate (i.e., thicker films should be obtained in ITO with the lowest sheet resistance). A noticeable change was noted for ITO type 3, where the reaction proceed faster and the obtained films had a large thickness and strong optical absorption, which exceeded the ellipsometer measurement range. However, it turned out that the obtained parameters did not have a significant impact on the PANI thickness films deposited onto ITO types 1 and 2. The results obtained for PANI clearly indicated a well-doped polymer with the presence of bands characteristic for polaronic/bipolaronic states.

### 3.5. Surface and Electrical Study

**Figure 6:**
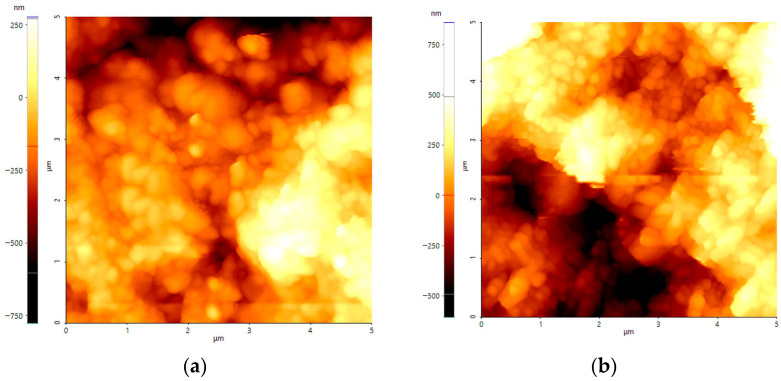
AFM images (5 µm × 5 µm) of PANI on ITO 70–100 Ω/sq (**a**) 7 cycles with 50 mV·s^−1^ and (**b**) 13 cycles with 100 mV·s^−1^.

Surface analysis was carried out using atomic force microscopy (AFM) (Park Systems XE 100, Suwon, Republic of Korea). Measurements were performed in two locations: in the central region of the sample (green area) and at the air–solution interface (dark green area, similar to blue). It was not possible to obtain measurements for the sample PANI on ITO 15–25 Ω/sq after seven cycles and at a scan rate of 50 mV·s^−1^ due to the layer being too thick (exceeding the measurement capabilities of the device). The AFM images, along with film thickness values and Root Mean Square Roughness (RMS), are summarized in Tables S8 and S9 and presented in the Supplementary Materials. The AFM images of PANI on ITO 70–100 Ω/sq prepared after seven cycles with 50 mV·s^−1^ and after 13 cycles with 100 mV·s^−1^ are shown in Figure 6.

**Figure 7:**
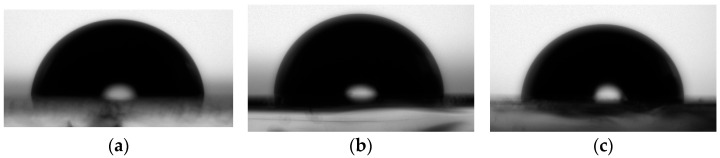
The drop of water on ITO (**a**) 70–100 Ω/sq, (**b**) 80–100 Ω/sq, and (**c**) 15–25 Ω/sq before washing.

The thickness values obtained in the central part of the sample were in good agreement with the ellipsometric calculations. The AFM images clearly show clusters of PANI agglomerates with a globular-shaped morphology, as seen in Figure 7, and this morphology is characteristic for PANI prepared from acidic conditions [70,88]. The process of creating conductive PANI films is well described in the work of Shishov M. A., where aniline oligomers are first absorbed on the ITO surface and self-assembled into clusters [89]. Next, clusters increase and turn into polymer spheres, and polymer spheres turn into granules according to the diffusion-limited aggregation [89]. The most conductive substrate yielded the highest film thickness, approximately 5.51 µm (although still lower than the values calculated in Section 3.1) as well as one of the highest RMS values. As mentioned before, overnight drying enhanced the mechanical durability of the coating. However, it ultimately led to film damage and an increase in the RMS roughness. For the remaining ITO substrates, lower film thicknesses and lower RMS values were obtained, which may indicate a more uniform electropolymerization process. The thickness of the layers near the air–solution interface ranged from 0.5 µm to 1.98 µm. At the air-solution interface, the formation of the PANI polymer is very rapid, leading to the formation of a partially oxidized form of PANI, and the thickness of the film may hinder the effective transport of ions in the polymer chain and between the granules [90].

Resistance measurements were performed using a four-point probe, based on which resistivity and conductivity were calculated. The formulas and results are presented in the Supplementary Materials (cf. Calculations for Section 3.5 and Table S7). The thickness of the layers for calculations were taken from ellipsometry and atomic force microscopy. The thickness of PANI obtained on ITO 15–25 Ω/sq was determined from the calculations presented in Section 3.1. The conductivity of PANI deposited on the ITO 15–25 Ω/sq substrate decreased significantly (below 0.051 S/cm) compared with the other substrates. This result confirms the presence of a mixture of emeraldine base and emeraldine salt in these layers, where charge transport is hindered also due to the increased thickness of the polymer film, as was mentioned above [90]. As demonstrated in [91], the conductivity of PANI films was heterogeneous, and enhanced conductivity in thicker films was attributed to the presence of highly-conductive domains. However, conductivity can decrease in thicker layers where poor film continuity and adhesion to the ITO substrate are observed. The same behavior can be seen in this case, where PANI conductivity is decreasing with increasing thickness, and larger differences also can be observed for ITO 70–100 Ω/sq substrates. It should be mentioned here again that the PANI layers were measured in air, which may also lead to additional oxidation and obtaining an oxidized form of PANI, reducing its conductivity [47,49]. The conductivity, calculated based on the thickness estimated by spectroscopic ellipsometry, of PANI on ITO 70–100 Ω/sq and 80–100 Ω/sq ranged from 0.622 S/cm to 12.924 S/cm, where the highest conductivity was calculated for PANI prepared on ITO 70–100 Ω/sq with 50 mV·s^−1^ and after seven cycles (cf. Table S7). The conductivity calculated using the AFM-determined thickness is similar to the conductivity calculated using the PANI thickness obtained from ellipsometry. The obtained conductivity is within the range given in the literature data [8,70,92,93].

As discussed in Section 3.3, an increased number of deposition cycles leads to a reduction in the optical energy band gap, which can be attributed to a higher contribution of polaronic states. In the case of electrical conductivity measurements, this reduction in the energy band gap as the number of deposition cycles increases (from 7 to 13 cycles) was accompanied by a decrease in electrical conductivity. However, for PANI deposited on ITO with a sheet resistance of 80–100 Ω/sq, the observed differences were relatively small, whereas for PANI on ITO with a sheet resistance of 70–100 Ω/sq, they were more pronounced (cf. the discussion above). Although the optical energy band gap values determined from Tauc plots varied depending on the electropolymerization parameters and the ITO substrate, establishing a direct and universal correlation between the optical band gap and the electrical conductivity is not straightforward. The electrical conductivity of polyaniline in its conducting form depends on the degree of protonation, morphology and efficiency of polaron/bipolaron charge transport [70].

Moreover, the water contact angle of glass/ITO substrates without pre-treatment (before washing in acetone and water) was determined and is presented in Figure 7. The contact angle for ITO 70–100 Ω/sq was 88°, for ITO 80–100 Ω/sq 89°, and for ITO 15–25 Ω/sq 90°. The obtained values indicate the more hydrophobic nature of ITO substrates [94].

The obtained contact angles were similar despite the ITO originating from different suppliers and exhibiting varying (or similar) sheet resistance. It is known that good adhesion between the ITO and the polymer results from strong intermolecular interactions. In the case of PANI, the reaction was conducted in an aqueous environment. Therefore, the ITO should exhibit the lowest possible contact angle, allowing for coverage of the entire ITO surface by penetrating micropores and irregularities, and enable the formation of uniform layers across the entire surface [95]. The water contact angle was measured again after sonification in acetone and water and decreased to the value of 76° for ITO 70–100 Ω/sq, 75° for ITO 80–100 Ω/sq and 74° for ITO 15–25 Ω/sq. In the future, the activation of ITO using plasma is planned, which will increase the hydrophilicity of the ITO layer.

## 4. Conclusions

Using an electropolymerization approach, the conducting form of PANI was deposited on three types of ITO substrates differing in surface resistivity. Aniline oxidation proceeded as an irreversible process, initiating the growth of PANI. The influence of the number of cyclic voltammetry cycles and the scan rate on the morphology and quality of the resulting PANI layers was systematically investigated. Comprehensive characterization was performed using ATR-IR spectroscopy, UV-Vis absorption, spectroscopic ellipsometry, AFM imaging, conductivity measurements, and water contact angle analysis.

For ITO with 15–25 Ω/sq, the fastest polymer growth and the greatest film thickness were observed. However, these layers exhibited poor adhesion and excessive optical absorption, which prevented reliable ellipsometric analysis. ATR-IR and UV-Vis spectroscopy confirmed the formation of a well-doped emeraldine salt and the presence of polaronic and bipolaronic states. The additional absorption maximum at 674 nm for PANI on ITO 15–25 Ω/sq indicates partial deprotonation to the emeraldine base, suggesting the presence of an ES/EB mixture. Ellipsometric modeling confirmed the expected optical behavior of the ITO substrates, which is in agreement with the manufacturer’s specifications. The highest conductivity was obtained for PANI on ITO 70–100 Ω/sq, whereas the lowest values were found for PANI on ITO 15–25 Ω/sq due to the mixed ES/EB composition, increased thickness, and reduced film continuity.

The synthesized PANI films on ITO with 70–100 Ω/sq and 80–100 Ω/sq exhibited a well-defined emeraldine salt structure for the applied number of cycles and scan rates together with an appropriate degree of protonation—features advantageous for bioelectronic and biological interfaces. In contrast, ITO substrates with very low sheet resistance (15–25 Ω/sq) led to excessively rapid electropolymerization, producing thick, non-uniform, poorly adhered, and only partially protonated films. Reducing the number of cycles may improve adhesion on these substrates. However, the overall results demonstrate that moderate sheet resistance provides the most favorable balance between the deposition rate, film quality, and final PANI conductivity.

Sulfuric acid doping enhances electrical conductivity and chemical stability, which are both essential for reliable biosensing performance. The optical and electrochemical features align well with literature values, indicating that the obtained materials fall within the expected range for biologically compatible PANI systems. Collectively, these findings suggest that ITO/PANI substrates are promising candidates for biological applications provided that the film uniformity and adhesion are adequately controlled.

## Figures and Tables

**Table 1:** Optical microscopy images of polyaniline (PANI) layers deposited on ITO substrates, showing surface morphology and non-uniformity (Nikon objective 10×/0.21 WD = 20.3 mm).

Number of Cycles/ Scan Rate	Polyaniline on ITO with:		
	70–100 Ω/sq Glass	80–100 Ω/sq Glass	15–25 Ω/sq Glass
7/ 50 mV‧s^−1^	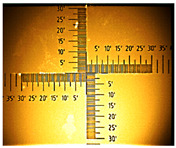	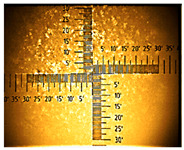	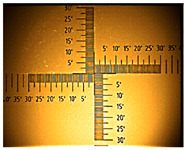
13/ 50 mV‧s^−1^	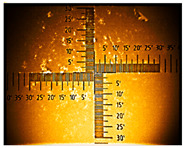	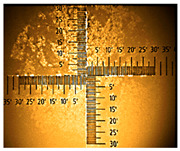	-
7/ 100 mV‧s^−1^	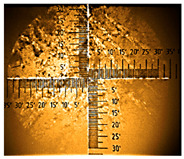	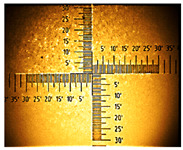	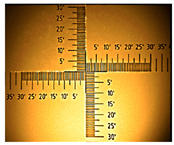
13/ 100 mV‧s^−1^	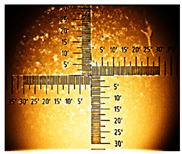	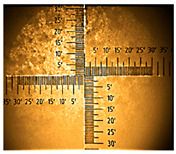	-

**Table 2:** The results obtained based on the ellipsometric Drude model parameters.

	ITO Type		
	70–100 Ω/sq	80–100 Ω/sq	15–25 Ω/sq
ω_p_ [1/s]	2.97·10^15^	2.98·10^15^	3.10·10^15^
τ [s]	7.33·10^−15^	6.48·10^−15^	8.11·10^−15^
μ [m^2^/V·s]	3.68·10^−3^	3.25·10^−3^	4.07·10^−3^
N [1/m^3^]	9.74·10^26^	9.75·10^26^	1.06·10^27^
σ [S/m]	5.75·10^5^	5.09·10^5^	6.89·10^5^
R [Ω/sq]	102.25	130.97	19.85

## Data Availability

Data are contained within the article and Supplementary Materials.

## Supplementary Materials

The following supporting information can be downloaded at https://www.mdpi.com/article/10.3390/nano16030165/s1, Table S1: Photo of the obtained polyaniline layers; Figure S1: Cyclic voltammograms obtained during the electropolymerization. The process was conducted in 0.5 mol·dm^−3^ H_2_SO_4_/water solution and the ITO working electrode with different surface resistivity. The scan rates were 50 mV·s^−1^ and 100 mV·s^−1^ and 7 or 13 cycles were performed; Figure S1. Continued: Cyclic voltammograms of the last cycle in 0.5 mol·dm^−3^ H_2_SO_4_/water solution; Figure S2: Plot of peak potential (E_p_^2a^ anodic and E_p_^1c^ cathodic [V]) vs. voltametric cycles—scan number [n]. Linear regression fitting was performed, and the coefficient of determination was calculated; Figure S3: Plot of the current [A] read at anodic (E_p_^a^) and cathodic (E_p_^c^) peak potentials obtained from CV voltammograms at different scan rates as a function of potential (V vs. Ag/AgCl); Figure S4: Plot of the current [A] read at anodic and cathodic peak potentials (current at E_p_^2a^ and E_p_^1c^) vs. voltametric cycles—scan number [n]. Linear regression fitting was performed, and the coefficient of determination was calculated; Figure S5: The proposed mechanism of the electrochemical synthesis of aniline on ITO based on [5,40,41,42,43,44,45,46,47,48,49,50,51,52,53,54,55]; Table S2: The areal and specific capacitance calculated for the last CV scan; Figure S6: ATR IR spectra; Figure S7: (a) Absorption spectra of PANI films on ITO 70–100 Ω/sq and 80–100 Ω/sq, and (b) absorption spectra of PANI on ITO 15–25 Ω/sq; Figure S8: (a) The ellipsometric model applied for ITO substrates modelling, and (b) the ellipsometric model applied for polyaniline films; Table S3: The maximum absorption bands (λ_abs_) with the energy band gaps; Figure S9: Optical and dielectrical coefficients obtained for the ITO substrates; Table S4: The optical and dielectrical coefficient values taken for λ = 1900 nm; Table S5: Drude parameters for ITO determined with ellipsometric model; Figure S10: (a) Optical coefficients n and (b) extinction k determined for PANI deposited on ITO substrates; Table S6: The resistivity (ρ) and conductivity (σ) calculation results; Table S7: AFM images (5 µm × 5 µm); Table S8: Thickness values of PANI, determined by AFM, with R_q_ and R_a_.
