# Supplementary material for: Aniline Electropolymerization on Indium–Tin Oxide Nanofilms with Different Surface Resistivity: A Comprehensive Study
Source: Nanomaterials (Basel). 2026 Jan 26;16(3):165. doi: 10.3390/nano16030165 (PMC12899808; doi:10.3390/nano16030165)
Supplement: Supplementary file 1 [file nanomaterials-16-00165-s001.zip › SM.pdf]

nanomaterials

MDPI

Article

Supplementary Material

Aniline Electropolymerization on Indium-Tin Oxide Nanofilms with Different Surface Resistivity: A Comprehensive Study

Sonia Kotowicz [1,*], Barbara Hajduk [2], Paweł Jarka [3], Agnieszka Katarzyna Pająk [2], Pallavi Kumari [2], Andreea Irina Barzic [4]

[1] Institute of Chemistry, Faculty of Science and Technology, University of Silesia, Szkolna 9 St., 40-006 Katowice, Poland.
[2] Centre of Polymer and Carbon Materials of the Polish Academy of Sciences, M. Curie-Skłodowkiej 43 St., 41-819 Zabrze, Poland; bhajduk@cmpw-pan.pl (B.H); apajak@cmpw-pan.pl (A.K.P.); pkumari@cmpw-pan.pl (P.K.)
[3] Department of Engineering Materials and Biomaterials, Faculty of Mechanical Engineering, Silesian University of Technology, Konarskiego 18A St., 44-100 Gliwice, Poland; pawel.jarka@polsl.pl
[4] "Petru Poni" Institute of Macromolecular Chemistry, Alea Grigore Ghica Vodă, no. 41A, 700487 Iaşi, Romania; cosutchi.irina@icmpp.ro
* Correspondence: sonia.kotowicz@us.edu.pl

Academic Editor: Firstname Lastname

Received: date
Revised: date
Accepted: date
Published: date

Citation: To be added by editorial staff during production.

Copyright: © 2025 by the authors. Submitted for possible open access publication under the terms and conditions of the Creative Commons Attribution (CC BY) license (https://creativecommons.org/licenses/by/4.0/).

List of contents:

Table S1. Photo of the obtained polyaniline layers……………………………………………..2
Figure S1. Cyclic voltammograms obtained during the electropolymerization. The process was conducted in 0.5 mol·dm^{-3} H_2SO_4/water solution, and the ITO working electrode with different surface resistivity. The scan rates were 50 mV·s^{-1} and 100 mV·s^{-1} and 7 or 13 cycles were performed……………………………………………………………………………………..2
Figure S1. Continued. Cyclic voltammograms of the last cycle in 0.5 mol·dm^{-3} H_2SO_4/water solution……………………………………………………………………………………..3
Figure S2. Plot of peak potential (E_p^{2a} anodic and E_p^{1c} cathodic [V]) vs voltametric cycles – scan number [n]. Linear regression fitting was performed and the coefficient of determination was calculated……………………………………………………………………………………4
Figure S3. Plot of the current [A] read at anodic (E_p^{a}) and cathodic (E_p^{c}) peak potentials obtained from CV voltammograms at different scan rates as a function of potential (V vs Ag/AgCl)……………………………………………………………………………………5
Figure S4. Plot of the current [A] read at anodic and cathodic peak potentials (current at E_p^{2a} and E_p^{1c}) vs voltametric cycles – scan number [n]. Linear regression fitting was performed and the coefficient of determination was calculated…………………………………………………6
Figure S5. The proposed mechanism of the electrochemical synthesis of aniline on ITO based on [5,40-55]……………………………………………………………………………………7
Calculations for *3.1. Electropolymerization*……………………………………………………7
Table S2. The areal and specific capacitance calculated for the last CV scan………………………8
Figure S6. ATR IR spectra……………………………………………………………………9
Figure S7. (a) Absorption spectra of PANI films on ITO 70 - 100 Ω/sq and 80 - 100 Ω/sq, and (b) absorption spectra of PANI on ITO 15 - 25 Ω/sq. ……..…………………………………………9
Figure S8. (a) The ellipsometric model applied for ITO substrates modelling, and (b) the ellipsometric model applied for polyaniline films……………………………………………………10
Table S3. The maximum absorption bands (λ_{abs}) with the energy-band gaps……………………10
Calculations for *3.3. Absorption study*…………………………………………………….11
Figure S9. Optical and dielectrical coefficients obtained for the ITO substrates…………………11
Table S4. The optical and dielectrical coefficient values taken for λ = 1900 nm…………………11
Table S5. Drude parameters for ITO, determined with ellipsometric model……………………12

Figure S10. (a) Optical coefficients n and (b) extinction k determined for PANI, deposited on ITO substrates……………………………………………………………………………………….12
Calculations for ***3.5. Surface and electrical study***…………………………………………………..12
Table S6. The resistivity (ρ) and conductivity (σ) calculation results……………….....................13
Table S7. AFM images (5 µm x 5 µm)…………………………………………………………………14
Table S8. Thickness values of PANI, determined by AFM, with R_q and R_a………………………16

Table S1. Photo of the obtained polyaniline layers.

Number of cycles / scan rate	Polyaniline, ITO with:		
	70-100 Ω/sq glass	80-100 Ω/sq glass	15-25 Ω/sq glass
7 / 50 mV·s^{-1}	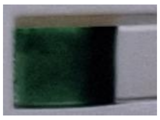	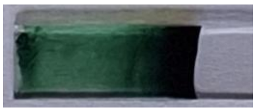	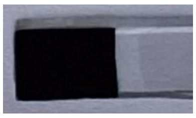
13 / 50 mV·s^{-1}	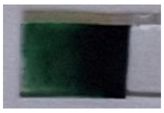	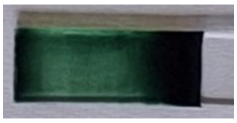	-
7 / 100 mV·s^{-1}	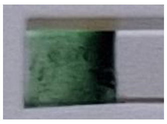	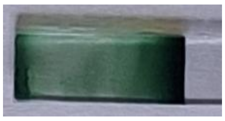	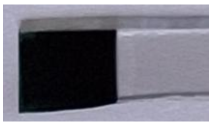
13 / 100 mV·s^{-1}	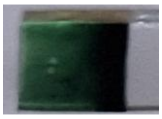	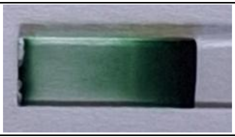	-

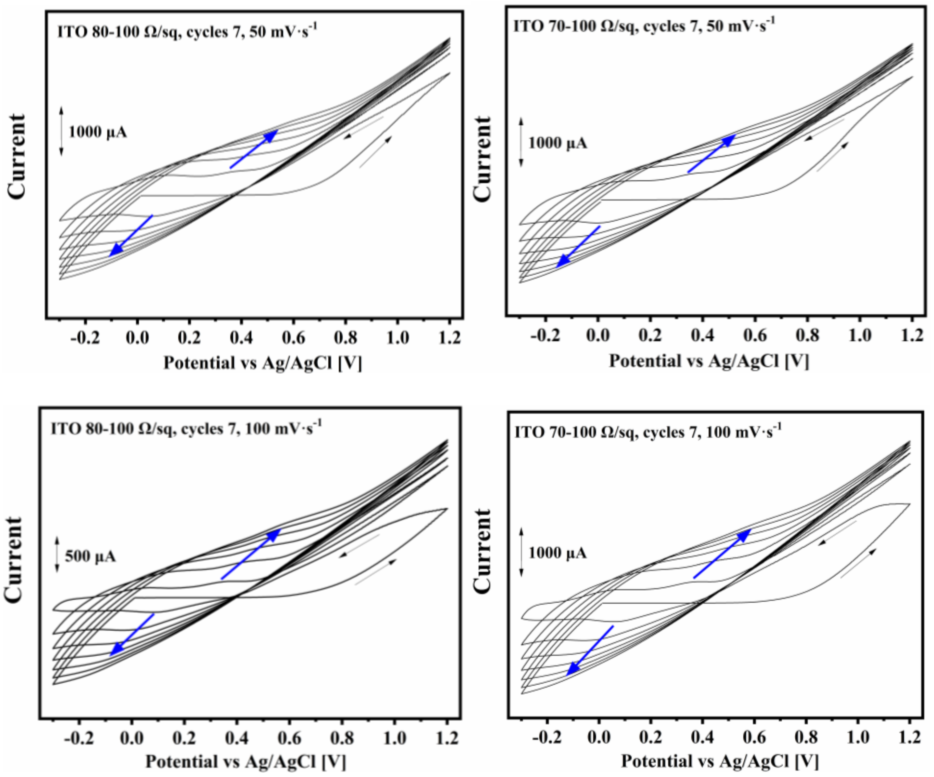

Figure S1. Cyclic voltammograms obtained during the electropolymerization. The process was conducted in 0.5 mol·dm^{-3} H_2SO_4/water solution with aniline, and the ITO working electrode with different surface resistivity. The scan rates were 50 mV·s^{-1} and 100 mV·s^{-1} and 7 or 13 cycles were performed.

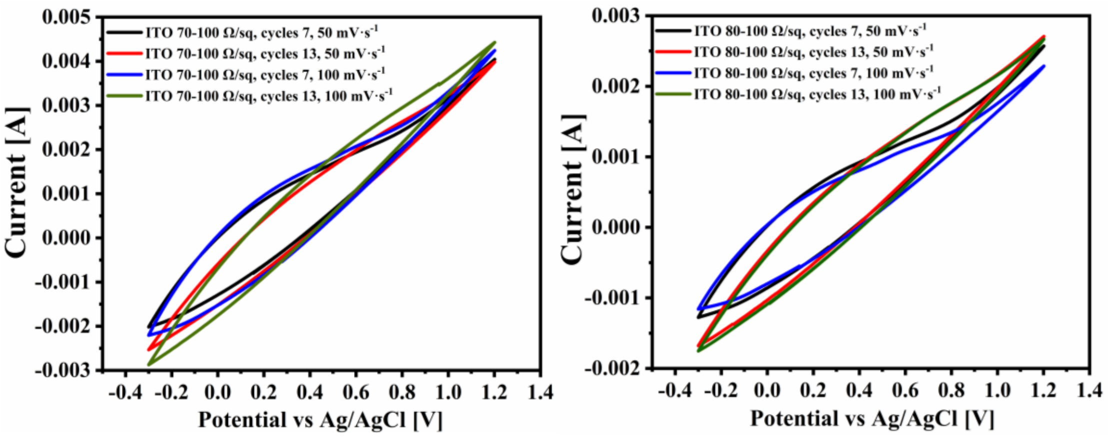

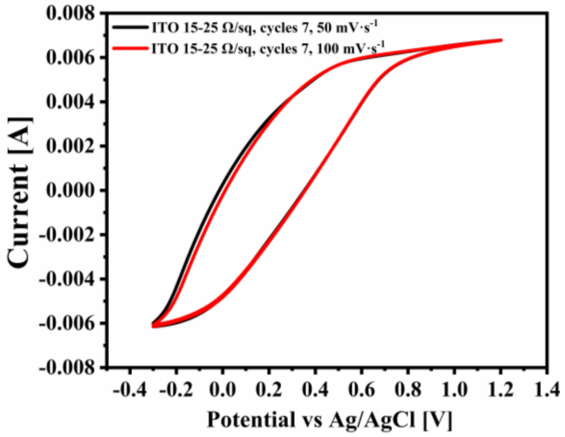

Figure S1. Continued. Cyclic voltammograms of the last cycle in 0.5 mol·dm^{-3} H_2SO_4/water solution.

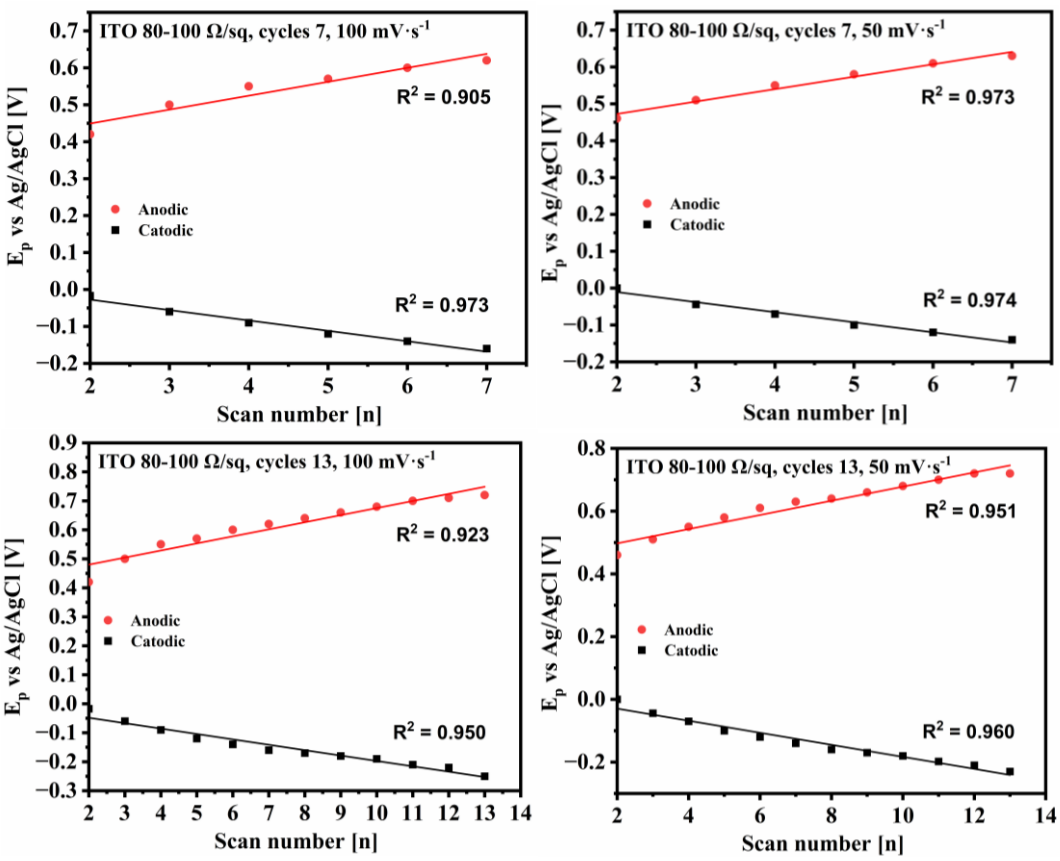

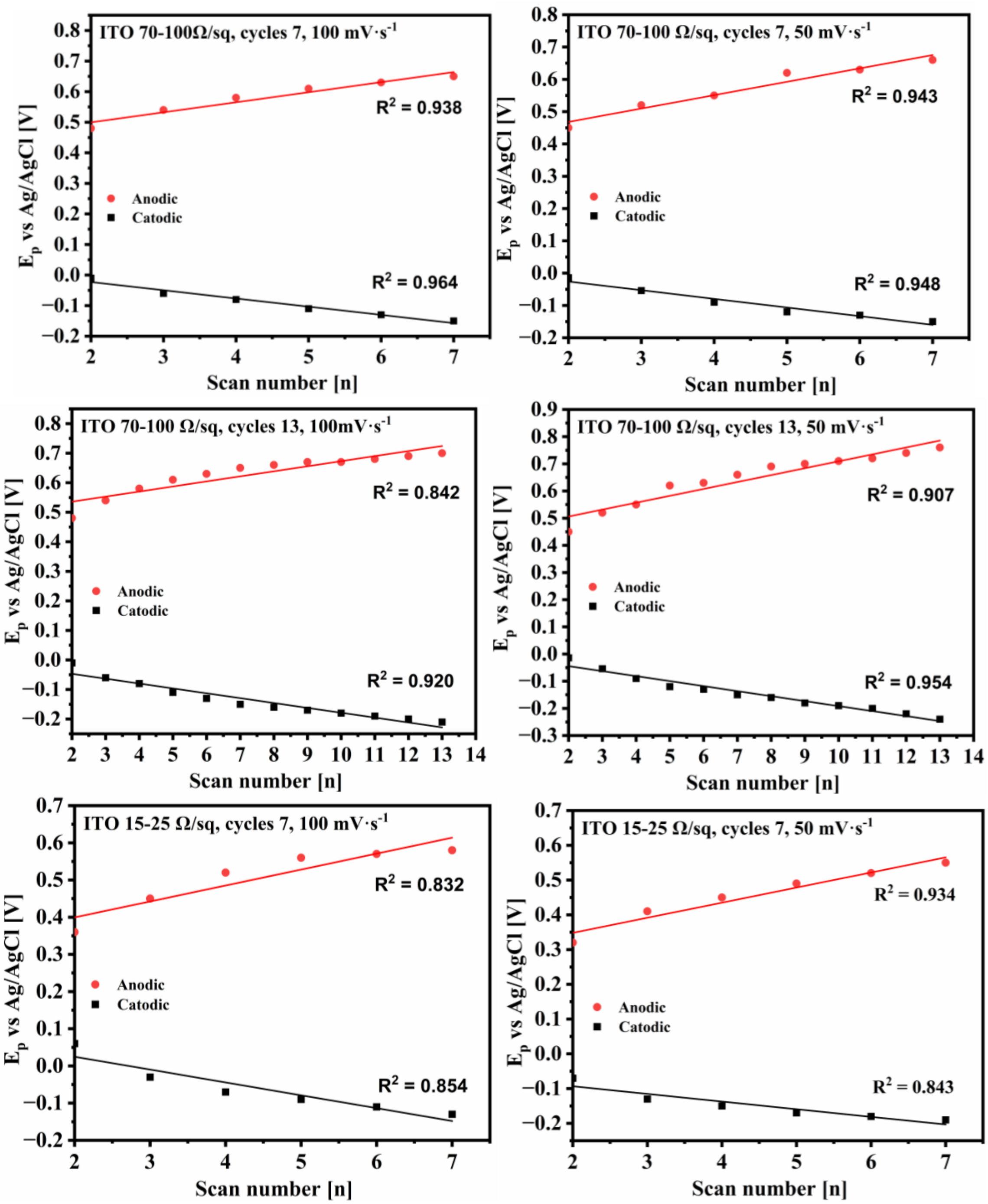

Figure S2. Plot of peak potential (E_p^{2a} anodic and E_p^{1c} cathodic [V]) vs voltametric cycles – scan number [n]. Linear regression fitting was performed and the coefficient of determination was calculated.

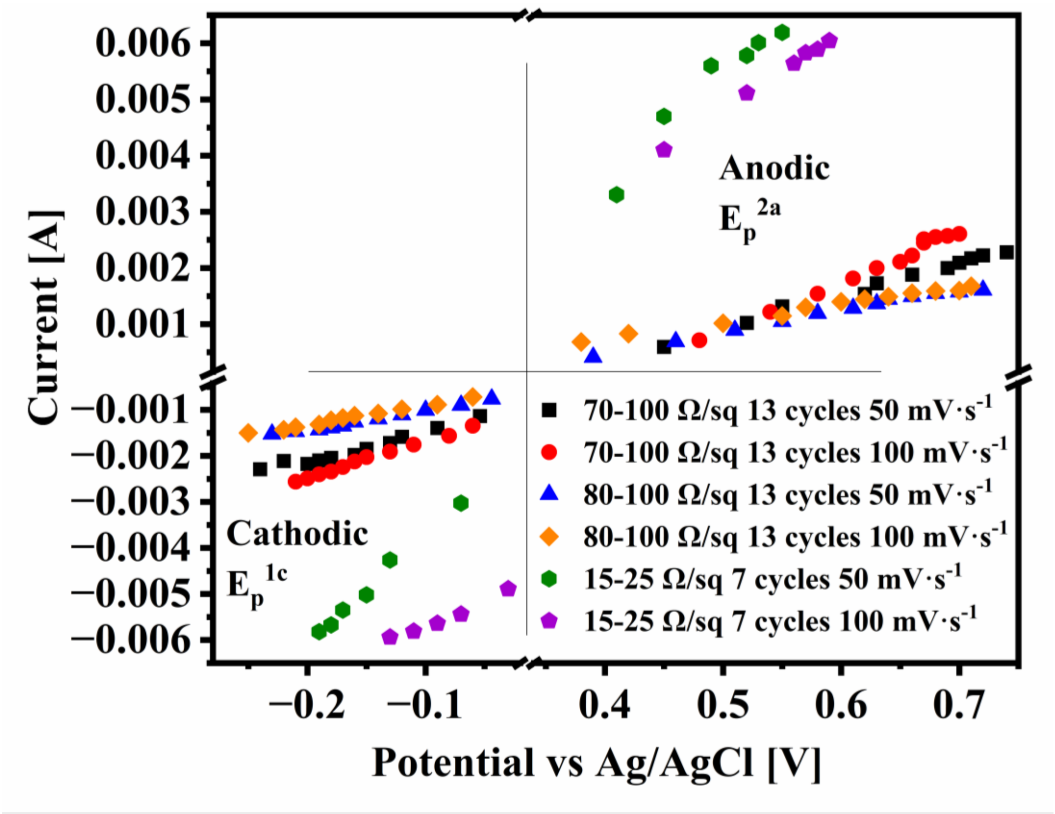

Figure S3. Plot of the current [A] read at anodic ($E_P{}^a$) and cathodic ($E_P{}^c$) peak potentials obtained from CV voltammograms at different scan rates as a function of potential (V vs Ag/AgCl).

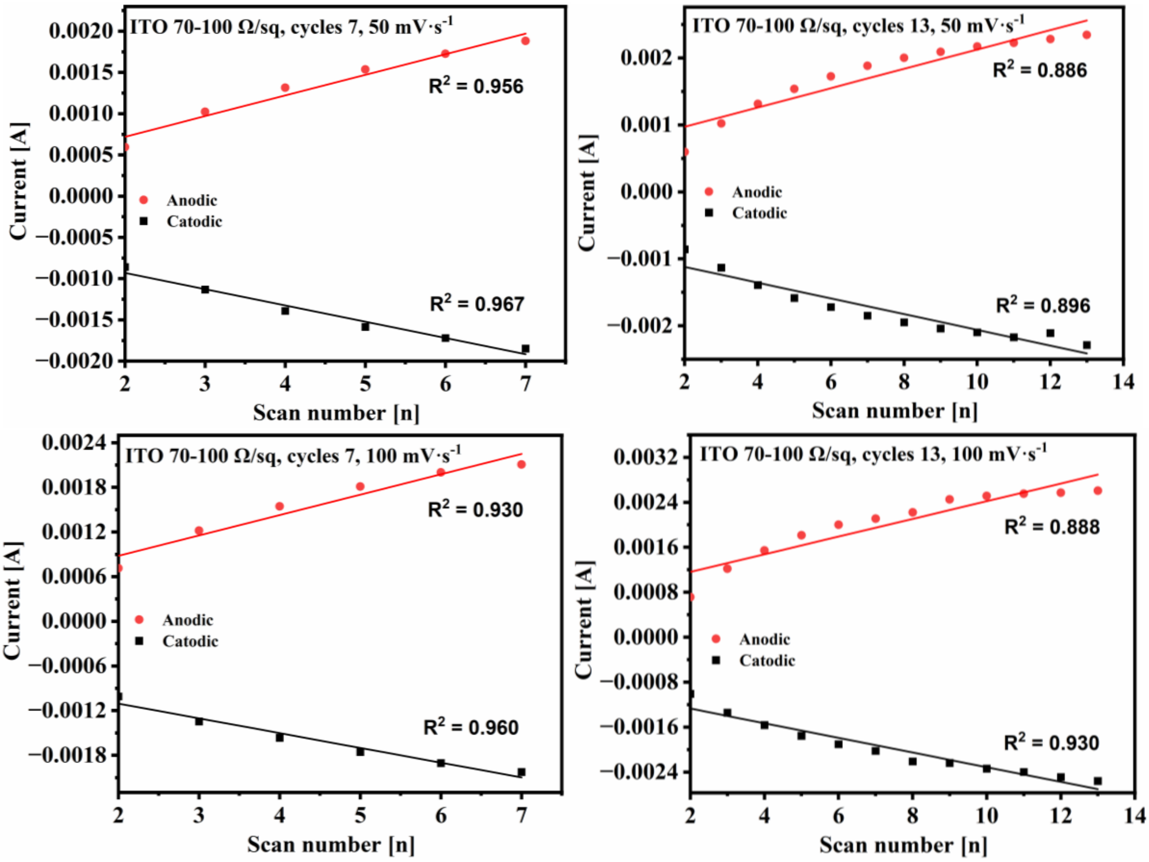

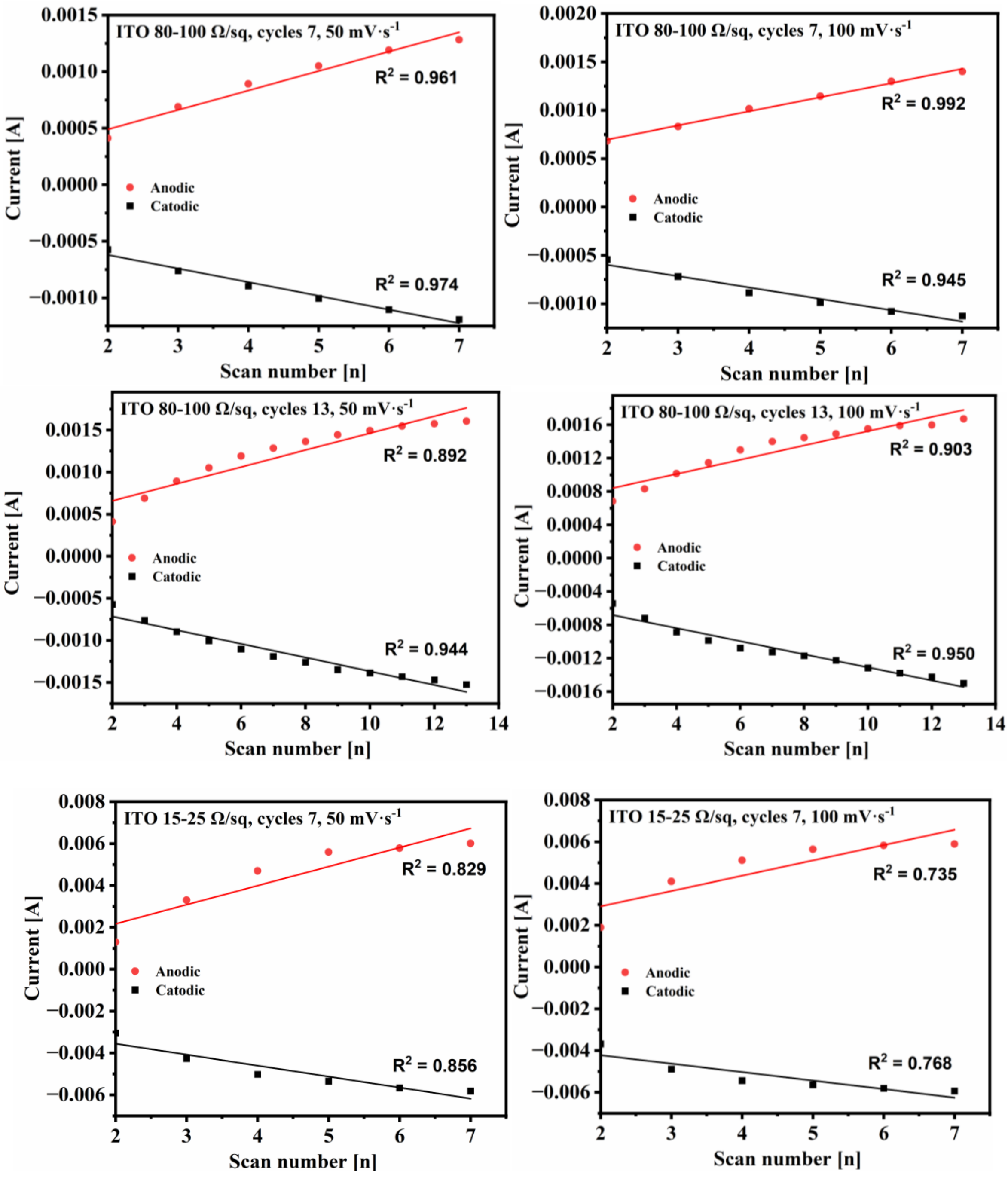

Figure S4. Plot of the current [A] read at anodic and cathodic peak potentials (current at E_P^{2a} and E_P^{1c}) vs voltametric cycles – scan number [n]. Linear regression fitting was performed and the coefficient of determination was calculated.

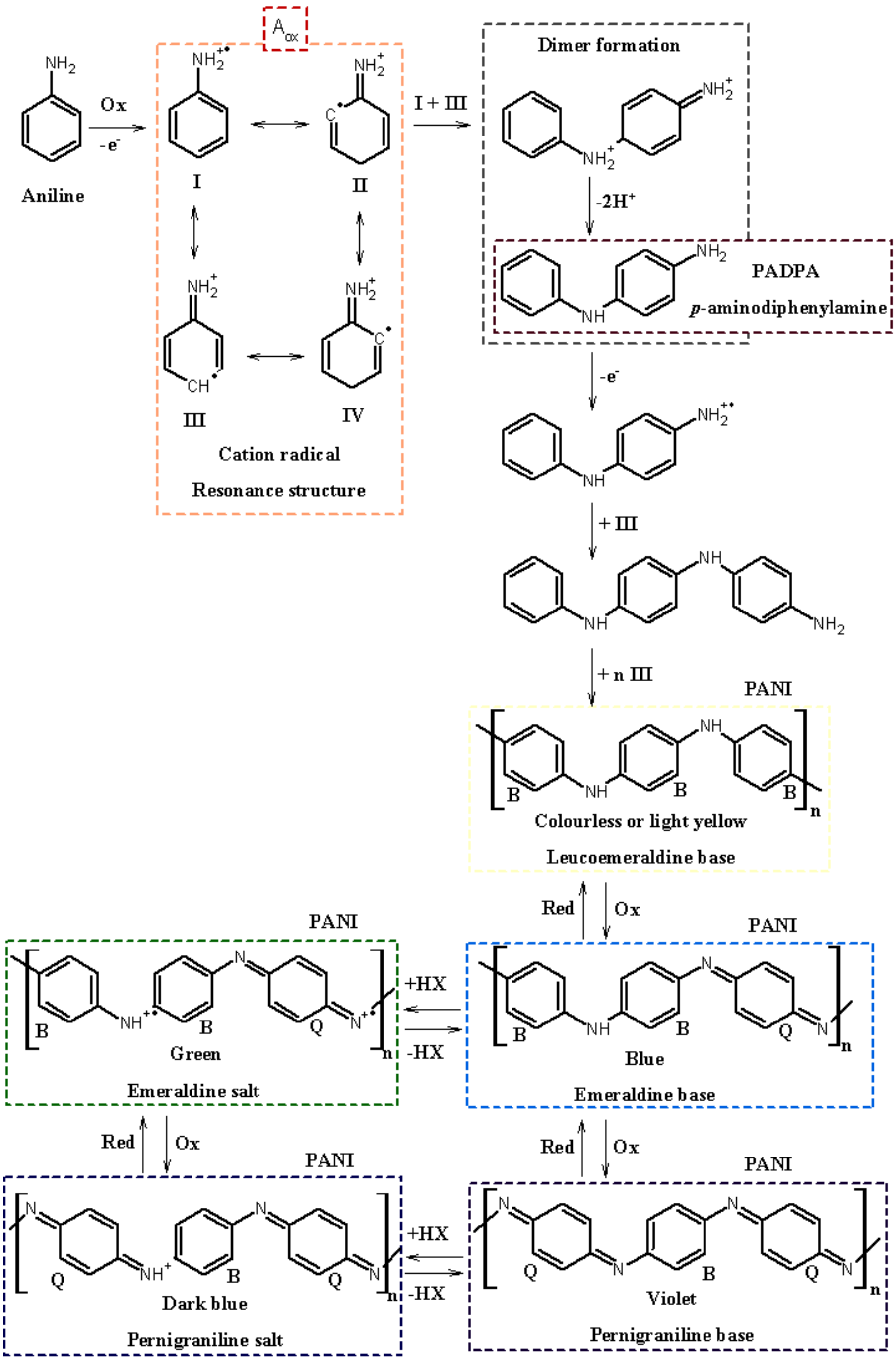

Figure S5. The proposed mechanism of the electrochemical synthesis of aniline on ITO based on [5,40-55].

Calculations for *3.1. Electropolymerization*:

➢ **Mass estimation based on Faraday's Law and [41]**

$$m = \frac{Q \cdot M}{z \cdot F}$$

where:

m – mass [µg],

Q – amount of charge calculated from the last cycle of CV [C],

M – molecular weight of the aniline monomer [g·mol^{-1}],

z – number of electrons,

F – Faraday's constants [C·mol^{-1}],

- **Thickness estimation based on [56;57;59;60]**

$$d = \frac{Q \cdot M}{z \cdot F \cdot S \cdot \rho}$$

where:

d	–	thickness [μm or nm],
S	–	working area of electrode [cm^2],
Q	–	amount of charge calculated from the last cycle of CV [C],
z	–	number of electrons,
F	–	Faraday's constants [$C \cdot mol^{-1}$],
M	–	molecular weight of the aniline monomer [$g \cdot mol^{-1}$],
ρ	–	specific density of aniline [$g \cdot cm^{-3}$].

- **Specific capacitance [58;61]**

$$C_{sp} = \frac{\int IdV}{2 \cdot m \cdot \vartheta \cdot \Delta V}$$

where:

C_{sp}	–	specific capacitance [$F \cdot g^{-1}$],
m	–	mass of the electroactive material [g],
$\int IdV$	–	integrated area under the CV curve from the last cycle [W],
ϑ	–	scan rate [$V \cdot s^{-1}$],
ΔV	–	potential window [V].

- **Areal capacitance [61]**

$$C_{ar} = \frac{\int IdV}{2 \cdot S \cdot \vartheta \cdot \Delta V}$$

where:

C_{ar}	–	areal capacitance [$F \cdot cm^{-2}$],
S	–	working area of electrode [cm^2],
$\int IdV$	–	integrated area under the CV curve from the last cycle [W],
ϑ	–	scan rate [$V \cdot s^{-1}$],
ΔV	–	potential window [V].

Table S2. The mass, areal and specific capacitance calculated for the last CV scan.

**Number of cycles / scan rate**	**m [g], C_{ar} [$F \cdot cm^{-2}$] and C_{sp} [$F \cdot g^{-1}$]; Polyaniline on ITO with:**		
	**70-100 Ω/sq glass**	**80-100 Ω/sq glass**	**15-25 Ω/sq glass**
**7 / 50 $mV \cdot s^{-1}$**	**m = $7.8 \cdot 10^{-6}$ g<br>C_{ar} = 16 $mF \cdot cm^{-2}$<br>C_{sp} = 575 $F \cdot g^{-1}$**	**m = $8.3 \cdot 10^{-6}$ g<br>C_{ar} = 11 $mF \cdot cm^{-2}$<br>C_{sp} = 599 $F \cdot g^{-1}$**	**m = $2.7 \cdot 10^{-5}$ g<br>C_{ar} = 33 $mF \cdot cm^{-2}$<br>C_{sp} = 997 $F \cdot g^{-1}$**
**13 / 50 $mV \cdot s^{-1}$**	**m = $7.9 \cdot 10^{-6}$ g<br>C_{ar} = 20 $mF \cdot cm^{-2}$<br>C_{sp} = 597 $F \cdot g^{-1}$**	**m = $8.5 \cdot 10^{-6}$ g<br>C_{ar} = 12 $mF \cdot cm^{-2}$<br>C_{sp} = 605 $F \cdot g^{-1}$**	**-**
**7 / 100 $mV \cdot s^{-1}$**	**m = $8.5 \cdot 10^{-6}$ g<br>C_{ar} = 8 $mF \cdot cm^{-2}$<br>C_{sp} = 565 $F \cdot g^{-1}$**	**m = $4.5 \cdot 10^{-6}$ g<br>C_{ar} = 5 $mF \cdot cm^{-2}$<br>C_{sp} = 515 $F \cdot g^{-1}$**	**m = $1.9 \cdot 10^{-5}$ g<br>C_{ar} = 28 $mF \cdot cm^{-2}$<br>C_{sp} = 1000 $F \cdot g^{-1}$**
**13 / 100 $mV \cdot s^{-1}$**	**m = $8.7 \cdot 10^{-6}$ g<br>C_{ar} = 9 $mF \cdot cm^{-2}$<br>C_{sp} = 585 $F \cdot g^{-1}$**	**m = $4.3 \cdot 10^{-6}$ g<br>C_{ar} = 6 $mF \cdot cm^{-2}$<br>C_{sp} = 568 $F \cdot g^{-1}$**	**-**

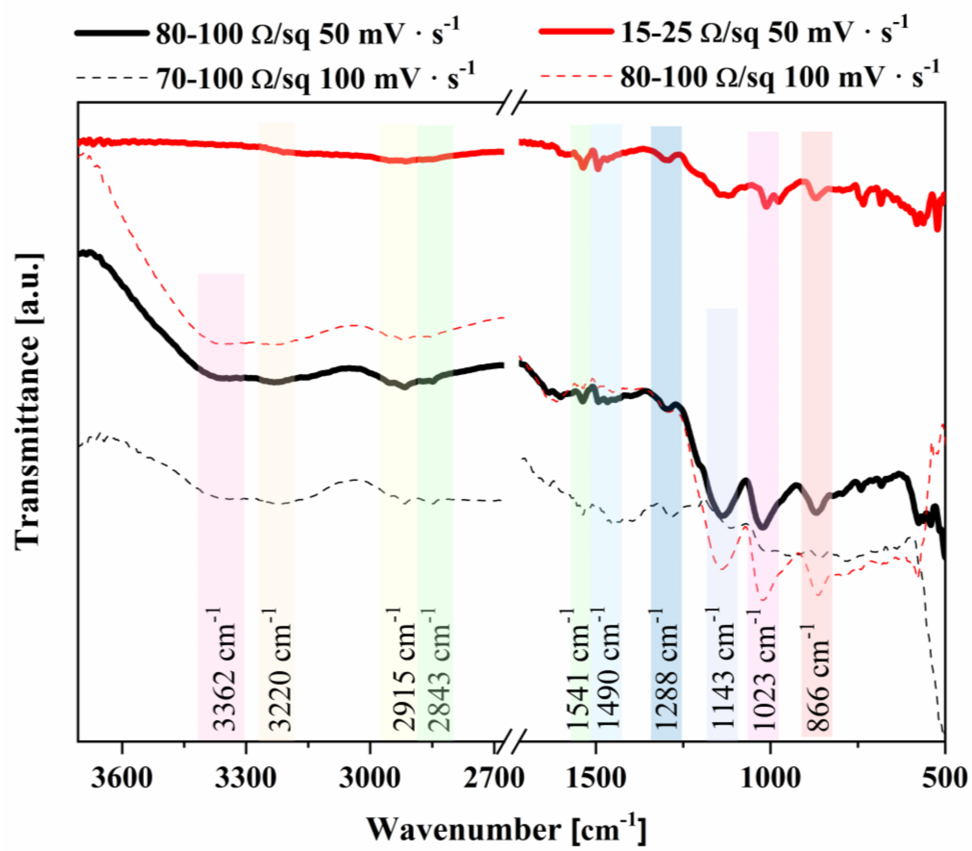

Figure S6. ATR IR spectra.

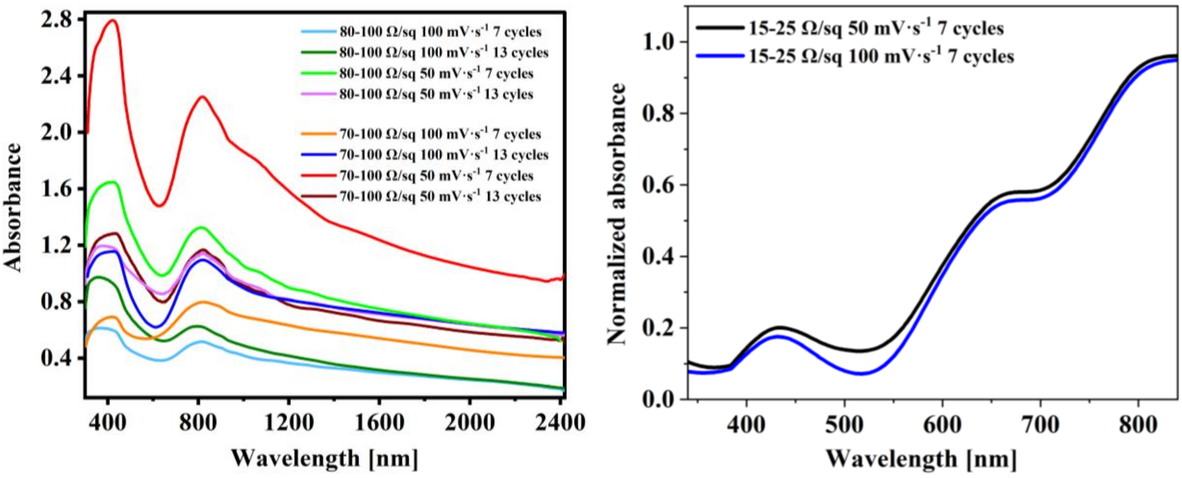

(a) (b)

Figure S7. (a) Absorption spectra of PANI films on ITO 70 - 100 Ω/sq and 80 - 100 Ω/sq, and (b) absorption spectra of PANI on ITO 15 - 25 Ω/sq.

Table S3. The maximum absorption bands (λ_{abs}) with the energy-band gaps (E_g).

**Number of cycles/ scan rate**	**λ_{abs} [eV] and [nm]; Polyaniline on ITO with:**		
	**70-100 Ω/sq glass**	**80-100 Ω/sq glass**	**15-25 Ω/sq glass**
**7 / 50 mV·s^{-1}**	**3.01; 1.51<br>412; 819<br>E_g^{DT} = 2.24 eV**	**3.46; 1.56<br>362; 794<br>E_g^{DT} = 2.34 eV**	**1.51; 1.84; 2.84<br>821; 674; 436<br>E_g^{DT} = n**
**13 / 50 mV·s^{-1}**	**2.91; 1.52<br>426; 815<br>E_g^{DT} = 2.31 eV**	**3.52; 1.56<br>352; 794<br>E_g^{DT} = 2.31 eV**	**-**
**7 / 100 mV·s^{-1}**	**3.02; 1.51<br>410; 819<br>E_g^{DT} = 2.34 eV**	**2.93; 1.52<br>423; 815<br>E_g^{DT} = 2.31 eV**	**1.51; 1.84; 2.83<br>821; 674; 438<br>E_g^{DT} = n**
**13 / 100 mV·s^{-1}**	**2.88; 1.51<br>430; 819<br>E_g^{DT} = 2.11 eV**	**3.29; 1.51<br>376; 819<br>E_g^{DT} = 2.17 eV**	**-**

E_g^{DT} – energy-band gap, direct transitions. n – couldn't get a proper fit.

(a)

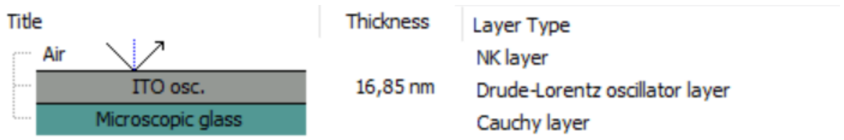

(b)

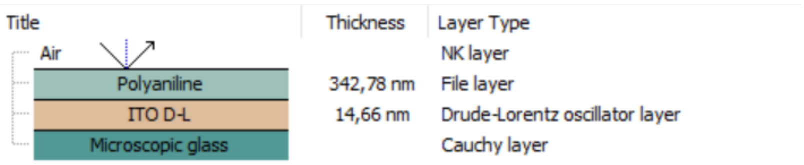

Figure S8. (a) The ellipsometric model applied for ITO substrates modelling, and (b) the ellipsometric model applied for polyaniline films.

Calculations for *3.3. Absorption study:*

➢ **Energy-band gap calculation based on the Tauc equation [78-81]:**

$$(\alpha h\nu)^{\frac{1}{n}} = A\,(h\nu - E_g)$$

where:

α	–	absorption coefficient,
h	–	Planck constant,
ν	–	frequency,
E_g	–	energy band gap,
A	–	proportionality constant,
n	–	Tauc exponent; n = $\frac{1}{2}$ direct transitions.

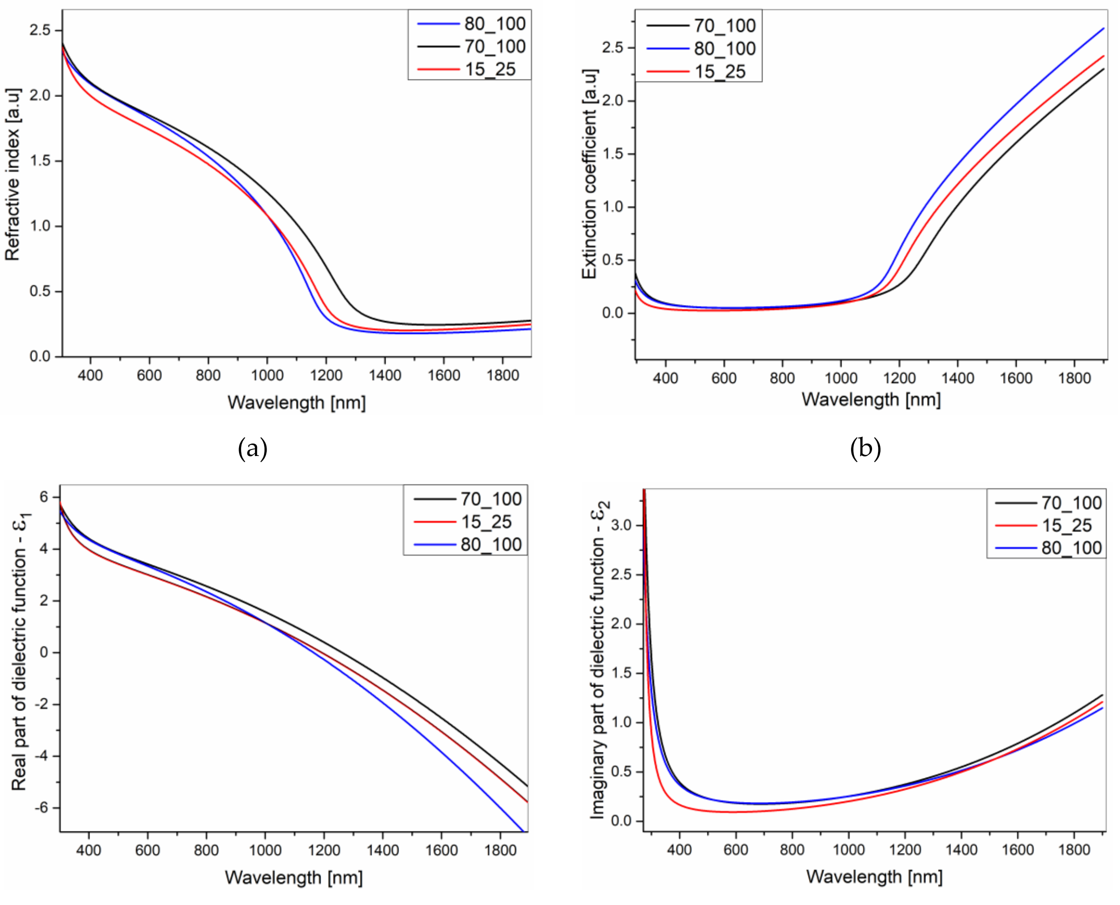

(a) (b)

(c) (d)

Figure S9. Optical and dielectrical coefficients obtained for the ITO substrates.

Table S4. The optical and dielectrical coefficient values taken for λ = 1900 nm.

**Parameter**	**ITO type**		
	**70-100 Ω/sq**	**80-100 Ω/sq**	**15-25 Ω/sq**
**n [a.u.]**	**0.28**	**0.22**	**0.25**
**k [a.u.]**	**2.30**	**2.69**	**2.42**

Table S5. Drude parameters for ITO, determined with ellipsometric model.

Parameter	ITO type		
	70-100 Ω/sq	80-100 Ω/sq	15-25 Ω/sq
Ω_p [1/cm]	15803	15810	16461
Ω_τ [1/cm]	724	819	655
d [nm]	17	15	73

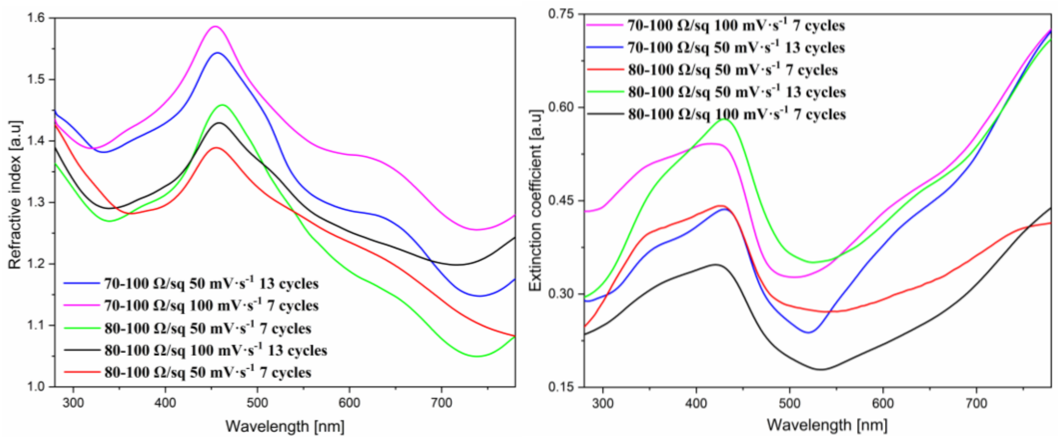

(a) (b)

Figure S10. (a) Optical coefficients n and (b) extinction k determined for PANI, deposited on ITO substrates.

Calculations for ***3.5. Surface and electrical study:***

➢ **Resistivity calculations for resistance measurement by four-point probe and s>d (s – distance between probes):**

$$\rho = \frac{U}{I} \cdot \frac{\pi \cdot d}{ln2}$$

where:

ρ – resistivity [Ω·cm],
d – thickness [cm],
$\frac{U}{I}$ – resistance [Ω].

➢ **Conductivity:**

$$\sigma = \frac{1}{\rho}$$

where:

ρ – resistivity [Ω·cm],
σ – conductivity [S/cm].

Table S6. The resistivity (ρ) and conductivity (σ) calculation results.

**Number of cycles/ scan rate**	**ρ [Ω·cm], σ [S/cm]; Polyaniline on ITO with:**		
	**70-100 Ω/sq glass**	**80-100 Ω/sq glass**	**15-25 Ω/sq glass**
**7 / 50 mV·s^{-1}**	$\rho_{elips.}$ = 0.077 ± 0.014<br>$\sigma_{elips.}$ = 12.924<br>ρ_{AFM} = 0.100 ± 0.014<br>σ_{AFM} = 9.995	$\rho_{elips.}$ = 0.698 ± 0.009<br>$\sigma_{elips.}$ = 1.432<br>ρ_{AFM} = 0.887 ± 0.009<br>σ_{AFM} = 1.127	$\rho_{cal.}$ = 19.624 ± 0.065<br>$\sigma_{cal.}$ = 0.0510
**13 / 50 mV·s^{-1}**	$\rho_{elips.}$ = 1.606 ± 0.015<br>$\sigma_{elips.}$ = 0.622<br>ρ_{AFM} = 1.768 ± 0.015<br>σ_{AFM} = 0.566	$\rho_{elips.}$ = 0.771 ± 0.023<br>$\sigma_{elips.}$ = 1.296<br>ρ_{AFM} = 0.898 ± 0.023<br>σ_{AFM} = 1.114	-
**7 / 100 mV·s^{-1}**	$\rho_{elips.}$ = 0.283 ± 0.012<br>$\sigma_{elips.}$ = 3.534<br>ρ_{AFM} = 0.278 ± 0.012<br>σ_{AFM} = 3.593	$\rho_{elips.}$ = 0.443 ± 0.046<br>$\sigma_{elips.}$ = 2.259<br>ρ_{AFM} = 0.516 ± 0.046<br>σ_{AFM} = 1.940	$\rho_{cal.}$ = 11.074 ± 0.075<br>$\sigma_{cal.}$ = 0.090<br>ρ_{AFM} = 6.102 ± 0.075<br>σ_{AFM} = 0.164
**13 / 100 mV·s^{-1}**	$\rho_{elips.}$ = 0.580 ± 0.031<br>$\sigma_{elips.}$ = 1.725<br>ρ_{AFM} = 0.464 ± 0.031<br>σ_{AFM} = 2.157	$\rho_{elips.}$ = 0.562 ± 0.054<br>$\sigma_{elips.}$ = 1.778<br>ρ_{AFM} = 0.598 ± 0.054<br>σ_{AFM} = 1.672	-

ρ – resistivity [Ω·cm], σ – conductivity [S/cm]. Measurement taken in the center part of the sample, positioning parallel to the four-point probe. Measurement performed three times. Elips. means calculation using thickness estimated by spectroscopic ellipsometry and AFM means calculation using thickness estimated by atomic force microscope. Cal. – resistivity and conductivity evaluated from the thickness calculations based on [56;57;59;60].

nanomaterials MDPI

Table S7. AFM images (5 µm x 5 µm).

**Number of cycles / scan rate**	**Polyaniline on ITO with:**		
	**70-100 Ω/sq glass**	**80-100 Ω/sq glass**	**15-25 Ω/sq glass**
**7 / 50 mV·s^{-1}**	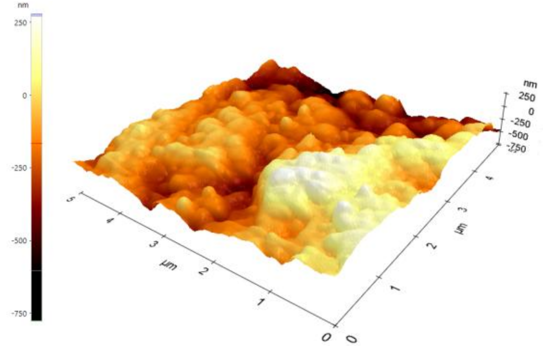	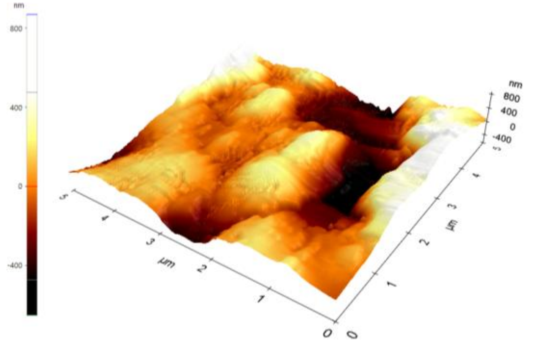	-
**13 / 50 mV·s^{-1}**	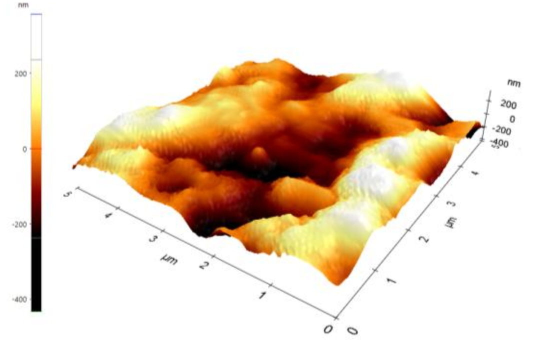	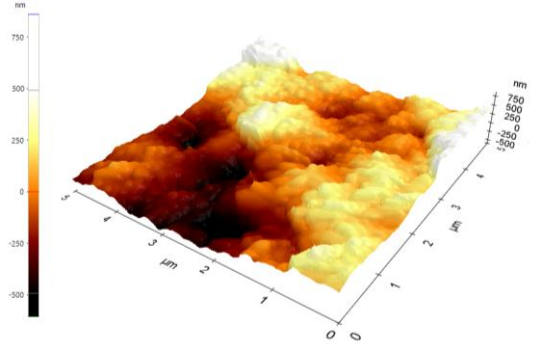	-

**7 / 100 mV·s^{-1}**	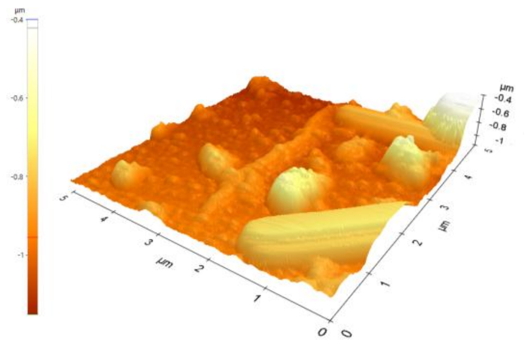	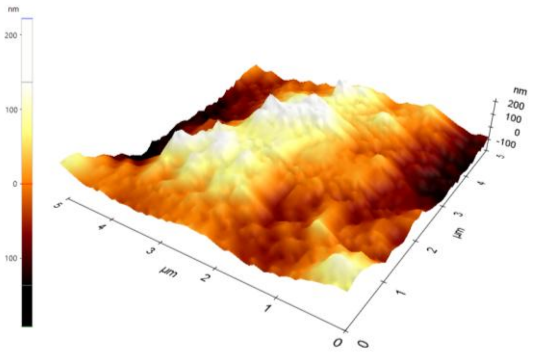	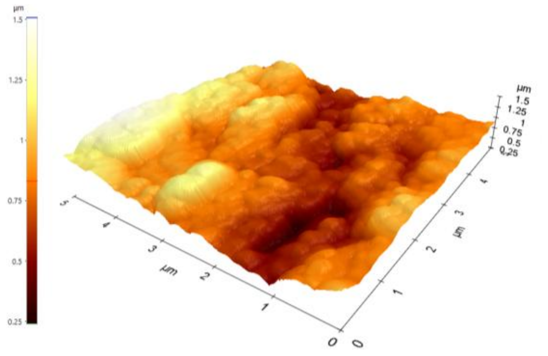
**13 / 100 mV·s^{-1}**	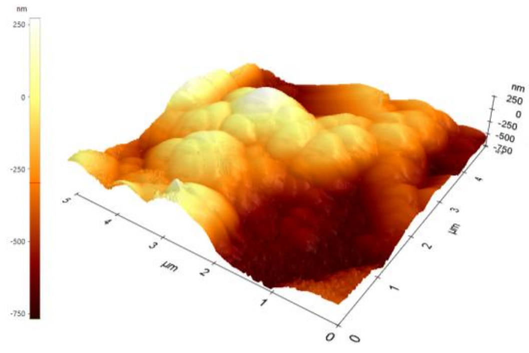	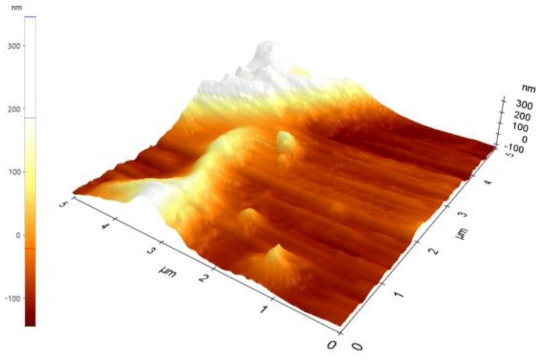	-

Table S8. Thickness values of PANI, determined by AFM, with R_q and R_a.

Number of cycles / scan rate	Polyaniline on ITO with:		
	70-100 Ω/sq	80-100 Ω/sq	15-25 Ω/sq
7 / 50 mV·s^{-1}	d* = 1 μm<br>d = 414 nm<br>R_q = 189 nm<br>R_a = 149 nm	d* = 1.72 μm<br>d = 631 nm<br>R_q = 718 nm<br>R_a = 568 nm	-
13 / 50 mV·s^{-1}	d* = 1.75 μm<br>d = 820 nm<br>R_q = 206 nm<br>R_a = 159 nm	d* = 1.98 μm<br>d = 881 nm<br>R_q = 318 nm<br>R_a = 254 nm	-
7 / 100 mV·s^{-1}	d* = 1.14 μm<br>d = 240 nm<br>R_q = 164 nm<br>R_a = 127 nm	d* = 0.50 μm<br>d = 396 nm<br>R_q = 94 nm<br>R_a = 75 nm	d = 5.51 μm<br>R_q = 625 nm<br>R_a = 532 nm
13 / 100 mV·s^{-1}	d* = 1.25 μm<br>d = 311 nm<br>R_q = 389 nm<br>R_a = 320 nm	d* = 0.98 μm<br>d = 654 nm<br>R_q = 290 nm<br>R_a = 216 nm	-

R_q – Root Mean Square (RMS) Roughness [nm], R_a – Roughness average [nm], d – thickness from the middle of the sample, green, d* – thickness close to the solution-air interface, dark green (a little bit of blue).
